# AutoBioTech—A Versatile Biofoundry for Automated Strain Engineering

**DOI:** 10.1021/acssynbio.4c00298

**Published:** 2024-07-08

**Authors:** Tobias Michael Rosch, Julia Tenhaef, Tim Stoltmann, Till Redeker, Dominic Kösters, Niels Hollmann, Karin Krumbach, Wolfgang Wiechert, Michael Bott, Susana Matamouros, Jan Marienhagen, Stephan Noack

**Affiliations:** † Institute of Bio- and Geosciences, IBG-1: Biotechnology, Forschungszentrum Jülich, D-52425 Jülich, Germany; ‡ Institute of Biotechnology, RWTH Aachen University, Worringer Weg 3, D-52074 Aachen, Germany; § The Bioeconomy Science Center (BioSC), Forschungszentrum Jülich, D-52425 Jülich, Germany

**Keywords:** *Escherichia coli*, *Corynebacterium glutamicum*, automation, modular cloning, CRISPR/Cas9

## Abstract

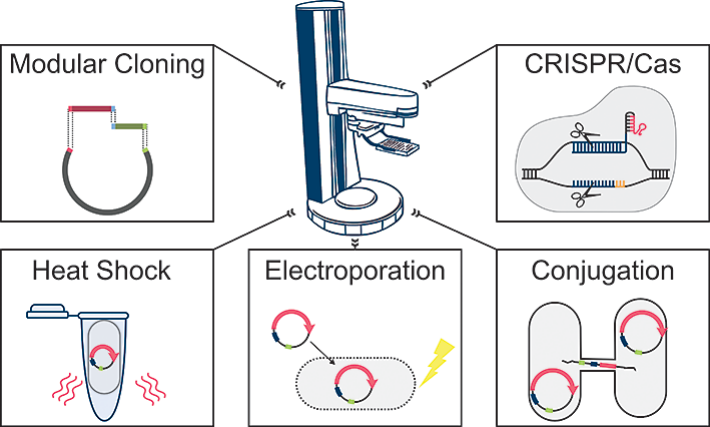

The inevitable transition from petrochemical production processes to renewable alternatives has sparked the emergence of biofoundries in recent years. Manual engineering of microbes will not be sufficient to meet the ever-increasing demand for novel producer strains. Here we describe the AutoBioTech platform, a fully automated laboratory system with 14 devices to perform operations for strain construction without human interaction. Using modular workflows, this platform enables automated transformations of *Escherichia coli* with plasmids assembled via modular cloning. A CRISPR/Cas9 toolbox compatible with existing modular cloning frameworks allows automated and flexible genome editing of *E. coli*. In addition, novel workflows have been established for the fully automated transformation of the Gram-positive model organism *Corynebacterium glutamicum* by conjugation and electroporation, with the latter proving to be the more robust technique. Overall, the AutoBioTech platform excels at versatility due to the modularity of workflows and seamless transitions between modules. This will accelerate strain engineering of Gram-negative and Gram-positive bacteria.

## Introduction

Laboratory automation in the field of biotechnology has made significant progress in recent years, revolutionizing the way experiments and research are conducted.^1^ While lab automation has often been pursued with a focus on a single task, e.g., for library preparation,^2^ there are increasing efforts to automate entire workflows in strain engineering.^3^ Emerging biofoundries around the globe offer automation and analytical capabilities to support this development.^4−6^ However, most of them do not work completely autonomously, but require manual material transfer between automation stations or offline process steps. Apart from these technical gaps, the realization of such “self-driving labs” also requires efficient data processing and management routines that enable integrated experimental design and hypothesis testing for strain engineering.^7^

Rational engineering of microbial producer strains for small molecules involves the (enhanced) expression of product-associated biosynthetic genes and the down-tuning or elimination of byproduct formation through targeted inactivation of genes involved in byproduct synthesis.^8−10^ To standardize and automate genetic engineering procedures, the preferred strategy is the modular cloning (MoClo) approach based on the Golden Gate cloning method.^11^ The latter is characterized by the utilization of Type IIS restriction enzymes, which cleave outside of their recognition site and produce a four-base overhang at the 5′-end. Strategic design of these overhangs enables the modular assembly and plasmid-based expression of functional transcription units (TU). Several MoClo libraries are available for various industrially relevant model organisms such as *Escherichia coli*, *Saccharomyces cerevisiae*, and *Corynebacterium glutamicum*.^12−14^

While MoClo is widely used to generate a multitude of DNA constructs in a combinatorial manner for numerous applications, the CRISPR/Cas-based methods have become the standard tools for precise genome editing.^15^ For example, the CRISPR/Cas9 system is used to introduce double-strand breaks into the chromosomal DNA of the target strain, which can be repaired, e.g., by homologous recombination.^16^ Using specifically designed templates, the resulting DNA sequence between homologous regions can be modified, including deletion and/or insertion of genomic sequences.^17^ Despite the inclusion of CRISPR/Cas9 components in the available MoClo kits, there is no application-specific, easy-to-implement methodology for automation.

Transformation of the Gram-negative bacterium *E. coli* with foreign DNA is a straightforward task via heat shock of chemically competent cells. In contrast, the cell envelope of the Gram-positive bacterium *C. glutamicum* makes the introduction of DNA into the organism much more challenging.^18,19^ Various transformation techniques for *C. glutamicum* have been developed, including protoplast and spheroplast transformation or phage-based transduction.^20^ However, the high number of medium additives and the high complexity of the workflows make these techniques unsuitable for automated high-throughput transformations.^21,22^ One technique for transforming *C. glutamicum*, that has been automated already, is conjugative DNA transfer using the *E. coli* S17–1 pEC-T18mob2 donor-plasmid system.^23−26^ A second technique, which to our knowledge has not yet been automated, is electroporation.^27,28^ For this method, plasmid size is an important factor, as it has been shown that larger plasmids can reduce the efficiency of electrotransformations.^29^ Moreover, in the case of *C. glutamicum*, the organism is exposed to a heat shock during transformations in order to increase transformation efficiency by up to 3 orders of magnitude.^30,31^ This is caused by a temporary inactivation of the native restriction-modification system.^32−34^

In this study, we present a new, versatile platform called “AutoBioTech” that enables the fully automated engineering of bacterial strains. The platform can automatically execute customized workflows for modular DNA assembly, on demand transformation, strain generation via CRISPR/Cas9-based genome editing, liquid and solid-media-based screening, as well as library preparation and screening for spectrophotometrically measurable properties. Compared to previously reported biofoundries, the AutoBioTech platform harmonizes these diverse capabilities in a single, autonomous robotic setup with a modular, application-independent and future-proof approach.^12,35−38^

## Results and Discussion

### Modular Platform for Automated Strain Engineering

**Figure 1:**
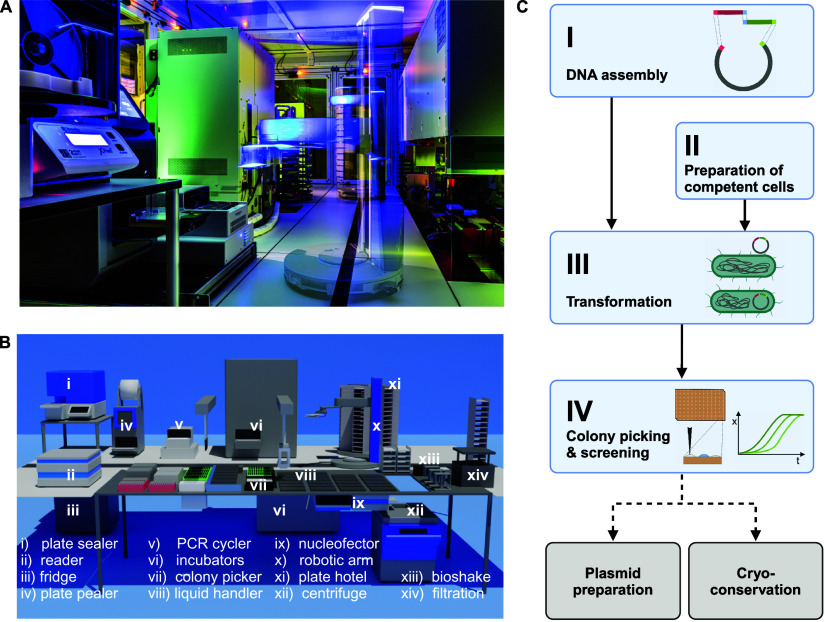
AutoBioTech platform for automated strain engineering. (A) Long-exposure photograph of the AutoBioTech platform, ©www.ahrens-steinbach-projekte.de. (B) Schematic representation of the platform, which includes a liquid handler, storage (hotel, carrousel) and incubation options (shaking incubator, incubator and fridge/freezer), a plate spectrophotometer (reader), a thermocycler (PCR cycler), a plate sealer and pealer. All devices are connected via a robotic arm on a bench track. Devices below the track (fridge, incubator) are accessed via transfer stations. (C) Basic workflow for construction of a plasmid-based strain library. Standardized basic (blue) and optional (gray) modules are concatenated to an automated workflow with material and data transfer between modules. Created with BioRender.com.

To tackle the challenges of faster and more robust strain development, standardization and automation of molecular cloning workflows are two key requirements. With the aim of creating a modular platform suitable for multipurpose strain engineering tasks, we have built up the AutoBioTech platform (Figure 1A,B). This platform combines a liquid handling system with devices for cultivation and incubation, for cooling, for polymerase chain reaction (PCR) using a thermal cycler and for screening using a plate spectrophotometer. For the transfer of labware in ANSI-SLAS format,^39^ all devices are connected via a robotic manipulator arm (“SCARA robot”) on a benchtop rail. Additionally, plates can be lidded or delidded as well as foil-sealed and desealed in an automated manner. The platform is housed in a HEPA-filtered enclosure to reduce contamination risks. Under the control of a scheduling software, experiments can be orchestrated in parallel with optimal use of equipment to reduce downtime. This enhances both productivity and reproducibility. For optimum use of space and to ensure expandability, larger devices are located under the bench track and made accessible via transfer stations.

**Figure 2:**
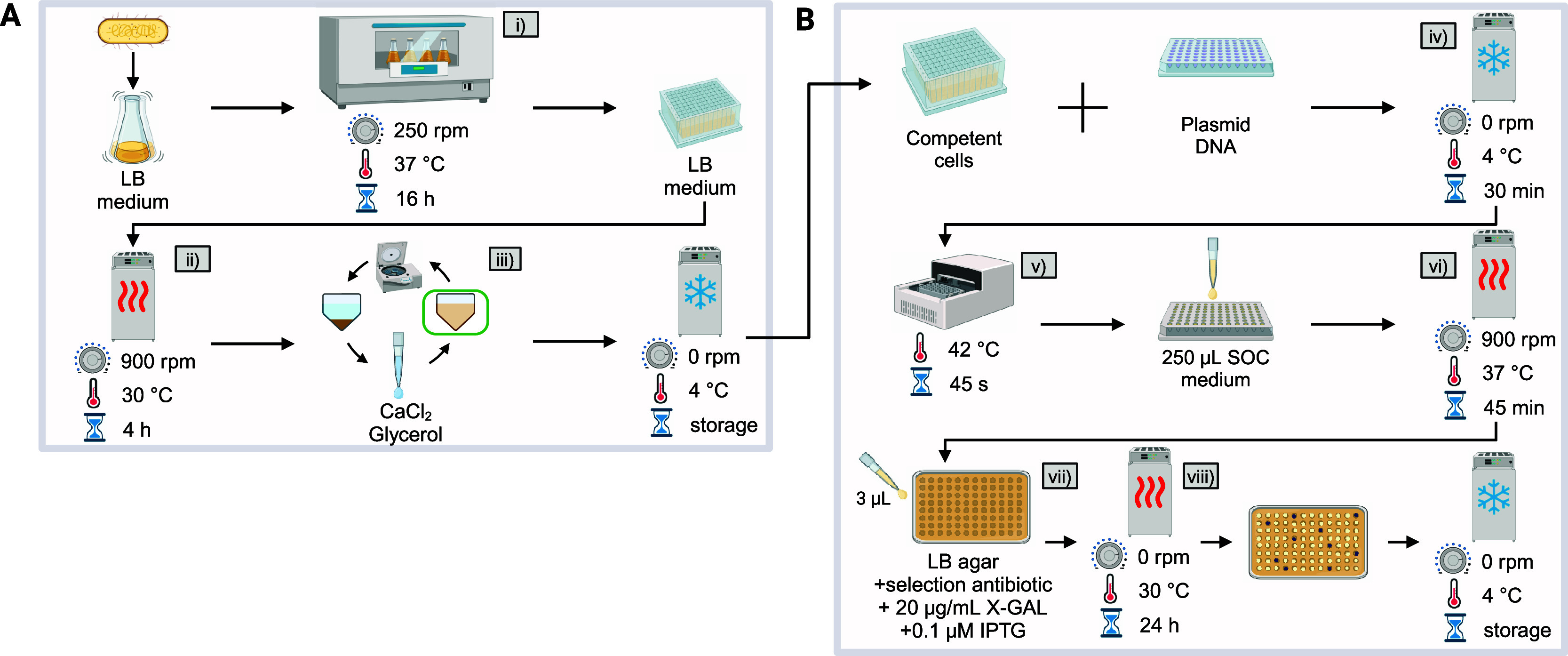
Automated transformation workflow for *E. coli*. (A) (i) Preculture, (ii) main culture, and (iii) competent cell generation. (B) (iv) incubation with DNA, (v) heat shock, (vi) transformant recovery, (vii) plating, and (viii) agar incubation. A detailed methodological description can be found in the Material and Methods section. Created with BioRender.com.

Using the AutoBioTech platform, several standardized modules have been developed that can be seamlessly combined into larger workflows or complemented by semiautonomous modules. The basic workflow for plasmid-based strain library construction of *E. coli* includes modular DNA assembly (Module I—Figure S1), preparation of chemically competent cells (Module II—Figure 2A), transformation followed by incubation on solid media (Module III—Figure 2B), colony picking and initial screening for growth and potential production (Module IV—Figure S2). Optional steps such as cryo-conservation of the generated strains and automated preparation of plasmids complete this workflow (Figure 1C).

### Basic Cloning Workflow for *E. coli*

**Figure 3:**
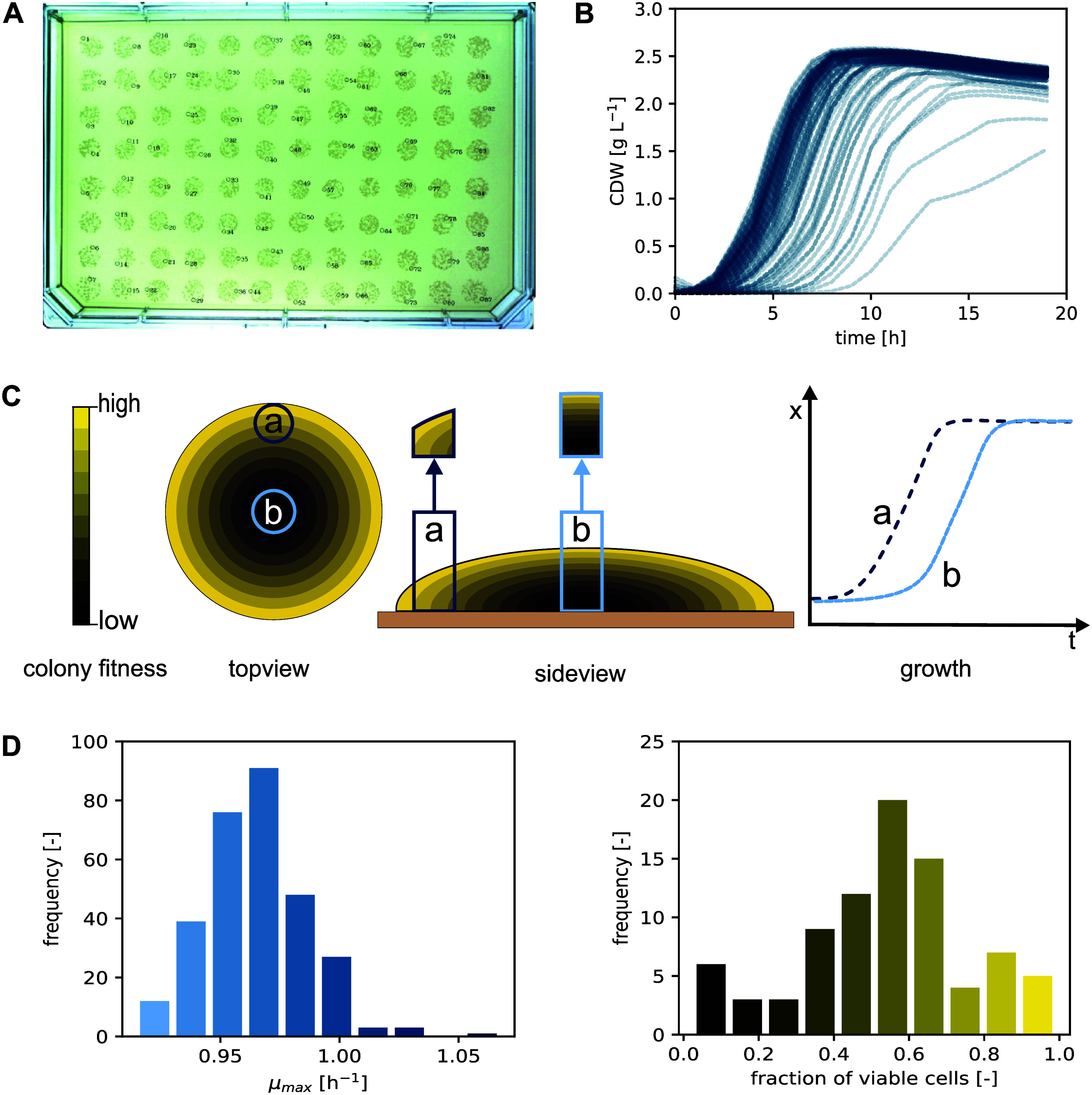
Automated molecular cloning of *E. coli*. (A) ANSI-SLAS agar plate showing colonies from 96 replicate transformations of *E. coli* DH5α with plasmid pRSET_B_JT. (Β) Growth profiles in LB medium of 87 picked colonies, picked from the agar in A. (C) Schematic of fitness heterogeneity in a single colony and the effect on consecutive biomass formation depending on the position and composition of picked colony fractions. (D) Estimated distributions of the maximum specific growth rate (left) and the fraction of viable cells in the inoculum (right).

To demonstrate the general functionality and efficiency of the AutoBioTech platform, the basic workflow was applied to transform *E. coli* DH5α with a midsized 3.6 kb plasmid based on pRSET_B (see Table S7 for details). In this first demonstrator, the automated DNA assembly step (Module I) was omitted in order to focus on transformation efficiency. Following the automated preparation of chemically competent cells (Module II), the transformation was carried out in 96 replicates. After spotting and incubating the transformants on an agar plate (Module III), colonies were obtained in all cases, corresponding to 100% transformation efficiency (Figure 3A). Finally, 87 isolated colonies were successfully picked, transferred to freshly prepared LB medium in a 96-well microtiter plate (MTP) and screened for growth (Module IV).

The cultures from picked colonies did show variation in growth pattern during the lag-phase (Figures 3B and S3) but most samples reached a similar maximum cell dry weight (CDW) of 2.59 ± 0.15 g L^–1^ in less than 12 h. The differences in the lag phase can result from variations in the total number of cells or a mixed population of viable and nonviable cells being picked from a single colony (Figure 3C). It has previously been shown, that growing and aging colonies develop heterologous metabolic states and fractions of alive and dead cells.^40^ To analyze the resulting growth phenotypes in more detail, a model-based approach was followed. In short, a two-species population model was formulated that takes into account potentially inactive cells in the initial biomass coming from a solid medium. In this way, the lag phase as a replicate-dependent property was decoupled from the maximum growth rate as a strain-specific parameter (see Supporting Information for details). As expected, the estimated maximum growth rates followed a narrow normal distribution with μ_max_ = 1.07 ± 0.02 h^–1^, while the proportion of initially active cells showed a larger variation, but was mainly below 60% (Figure 3D).

Despite variations in biomass production, this first demonstrator proved the successful automation of the transformation, colony picking and screening (Modules II–IV) in a 96-well format as key elements for rational strain engineering with the AutoBioTech platform.

### Modular Cloning (MoClo) for Plasmid-Based Strain Libraries

As a first step toward high-throughput strain engineering, the readily available CIDAR MoClo kit for *E. coli* was applied in the AutoBioTech platform.^13^ This cloning system allows for the flexible assembly of transcription units (TUs) with one or more genes by utilization of standardized genetic elements such as promoters, ribosome binding sites (RBS) and terminators.

**Figure 4:**
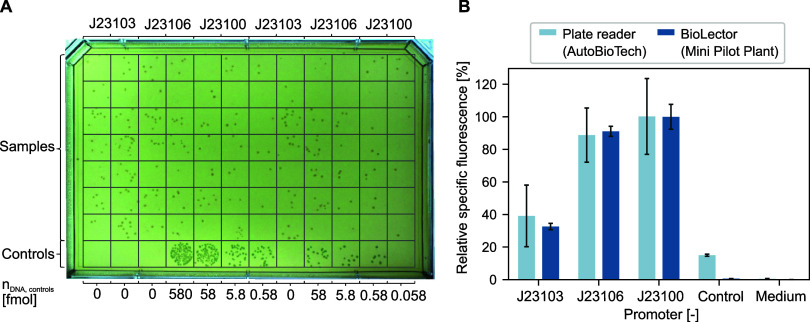
Automated generation of plasmid-based strain libraries of *E. coli*. (A) SBS agar plate displaying colonies from *E. coli* DH5α carrying *gfp*-containing plasmids under the control of three different constitutive promoters (J23103, J23106, and J23100). Rows 1–7: MoClo transformants containing the promoter as indicated above the plate picture. Row 8: negative and positive controls transformed with different amounts of empty vector as indicated below the plate picture. (B) Phenotyping of transformants with the three different promoters. *E. coli* DH5α lacking the plasmid vector or with the empty vector DVK_AE was used as negative and positive control, respectively. The specific GFP fluorescence is depicted after cultivating cells for 24 h in LB medium containing 50 μg mL^–1^ kanamycin and was measured in a plate reader as well as the BioLector Pro.

To test the modular DNA assembly combined with the automated transformation, colony picking and phenotyping capabilities of the AutoBioTech platform, assemblies of TUs from four DNA parts were conducted. *gfp* for green fluorescent protein (GFP) production as the gene of interest was assembled with three different promoters J23103, J23106, and J23100 with increasing strength,^13^ the RBS BCD2 and the terminator B0015 in vector DVK_AE (see Table S3). This vector allowed for blue-white screening due to the *lacZ*α fragment in the cloning site AE. Direct transformation of MoClo reactions into *E. coli* DH5α using the pre-established automated routine at the AutoBioTech platform showed that most (85.7%) reactions yielded transformants, of which all were regarded to carry successfully assembled plasmids according to the blue-white screening (Figure 4A). As expected, negative controls lacking the plasmid vector did not form colonies and positive controls with the empty vector DVK_AE showed an increasing number of colonies with increasing amount of employed DNA.

Screening in liquid medium for the production of GFP of at least six transformants per promoter-variant were conducted to test whether the *gfp* gene had been introduced into the plasmids. Specifically, the end-point specific fluorescence for each culture was calculated from absolute OD_600_ and fluorescence intensity measurements performed with the integrated plate reader (Figure 4B) and a MTP with 100 μL culture volume per well. After normalization to the maximum mean-fluorescence, J23100 and J23106 resulted in the highest relative specific fluorescence of 100% and approximately 90% respectively, while J23103 resulted in approximately 39%, resembling previously described promotor performance.^13^ These results were confirmed by an independent phenotyping experiment using the established Mini Pilot Plant technology, which allows well-controlled cultivation in MTPs and online measurement of backscatter and fluorescence.^41^ In this setup, the same trend was observed, which indicates comparability between these two phenotyping systems. Furthermore, for each promoter at least three colonies were used for plasmid preparation and sequencing using primers P1 and P2 (see Table S6) to test for correct plasmid assembly. This revealed a 100% identity of the expected and actual sequence after cloning.

### Combining MoClo and CRISPR/Cas9 for Automated Genome Engineering

**Figure 5:**
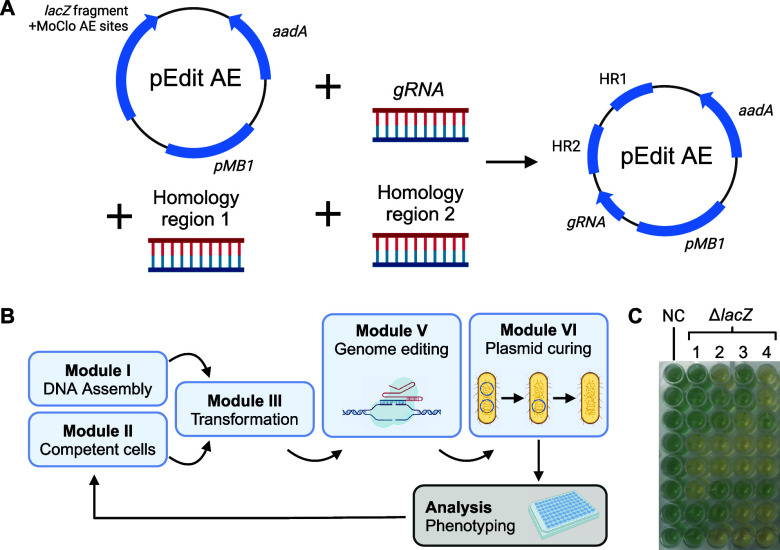
Automated genome engineering of *E. coli* combining MoClo and CRISPR/Cas9. (A) Assembly of plasmid pEdit_AE with three DNA parts. *pMB1*: origin of replication, *aadA*: aminoglycoside (3″) (9) adenylyltransferase/spectinomycin resistance gene, HR: homology regions for genomic recombination, *gRNA*: variable gRNA sequence. (B) Iterative AutoBioTech workflow covering the transformation of prepared cells with pEdit plasmid, genome editing with CRISPR/Cas9, as well as plasmid curing and analysis. (C) Phenotyping of edited transformants; NC: negative control, 1–4: pEdit1–4; blue coloring shows β-galactosidase activity indicating unsuccessful editing. Created with BioRender.com.

Jiang et al. introduced a two-plasmid design for CRISPR/Cas9-based genome editing of *E. coli*.^16^ The first plasmid “pCas” contains the genes for the Cas9 nuclease, the λ-red system, a temperature-sensitive origin of replication, and a gRNA targeting the second plasmid (Figure S4). The second plasmid “pEdit” contains the specific homologous regions for the homologous recombination and encodes the gRNA that targets the genome. The latter was created by exchanging the Cas9 guiding region in plasmid pTarget (see Table S7) for the *lacZ* fragment with sites AE from plasmid DVK_AE (see Table S3), which made the new plasmid, pEdit, MoClo compatible (Figure 5A). In this way, a standardized genome editing system for *E. coli* was developed, which was integrated into a fully automated workflow with the help of the AutoBioTech platform (Figure 5B). Several rounds of genome editing are possible until the final curing of pCas plasmid is initiated (Figure S4).

To test the established two-plasmid system, it was aimed to delete parts of the *lacZ* gene in *E. coli* MG1655. For this, different versions of the plasmid pEdit were manually created containing three different gRNAs and two different combinations of homologous regions per gRNA (see Tables S5 and S6). The resulting plasmids pEdit 1, 3, and 5 would delete 3021 bp of the 3075 bp large *lacZ* gene, while plasmids pEdit 2, 4, and 6 would delete 1500 bp, 1176 bp and 1373 bp, respectively. Notably, transformation of *E. coli* MG1655 pCas with plasmids pEdit 5 and 6 was not possible as no transformant colonies could be generated. Possibly, the spacer used for these plasmids (see Table S6) caused unspecific targeting of the Cas9 protein, which could have led to increased cell mortality. Although this theory could not be verified, the observed effect implies that currently available computational methods are incapable of predicting robustly functional gRNA sequences.

Regardless of this effect, pEdit 1–4 could be used for automated genome editing. *lacZ* deletion was analyzed phenotypically by testing for the ability to hydrolyze X-gal and release the blue-colored substituted indole (Figure 5C). This approach, with 8 replicates for each version of pEdit, revealed a success rate of 56.3%. Since only a single colony was picked from each replicate, it is highly likely that repeated picking could improve the success rate of the workflow. Nevertheless, it was demonstrated that the developed modular genome editing system is capable of deleting portions in the genome of *E. coli* and could become applicable for production-oriented editing.

### Automated Transformation of *C. glutamicum*

The strain engineering workflow described above is inapplicable to *C. glutamicum*, which is the second organism targeted by the AutoBioTech platform. To enable the automated transformation of this bacterium, high-throughput conjugation and electroporation were employed.

The former method was previously described by us in a semiautomated environment but autonomous strain engineering requires full automation of each module. For this reason, parts of the conjugation workflow were successfully scaled down to use automation-compatible SBS labware.^23^ A detailed description of workflow optimization to increase conjugation efficiencies is provided in the Supporting Information. In short, using an adapted heat shock duration (HSD) of 3 min, conjugation efficiencies of approximately 100 CFU mL^–1^ (for calculation see eq 8 in Supporting Information) could be reached for two plasmids.

**Figure 6:**
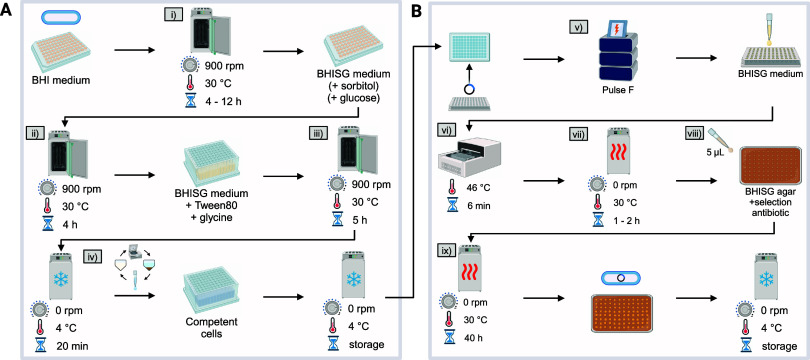
Automated electroporation workflow for *C. glutamicum*. (A) (i) Preculture, (ii) second preculture, (iii) main culture and (iv) competent cell generation. (B) (v) Electroporation, (vi) heat shock, (vii) transformant recovery, (viii) plating, and (ix) agar incubation. A detailed methodological description can be found in the Material and Methods section. Created with BioRender.com.

Electroporation was investigated as an alternative method for the transformation of *C. glutamicum*. Until now, this could not be automated for bacteria, but with the recent release of the 4D-Nucleofector (Lonza, Basel, Switzerland), automated electroporation in a 96-well format became possible. By integrating this device into the AutoBioTech platform, a fully automated workflow was developed enabling up to 92 parallel, independent transformations of *C. glutamicum* (Figure 6).

Preliminary manual tests with the 4D-Nucleofector showed that a 100% success rate of transformations with an average efficiency of 1082 ± 504 CFU μg^–1^ or approximately 2000 CFU mL^–1^ can be achieved with plasmid pEC-T18mob2_*ptuf-eYFP* (Figure S9 and Table S7). However, one phenomenon that occurred after automated filling of the 96-well Nucleocuvette Plate was arcing in 73.9% of all filled wells.^42^ The result were fewer or no viable transformants in wells where arcing occurred. Arcing in these wells likely stemmed from the introduction of air bubbles, as an improved liquid handling to avoid pipetting air reduced the rate of arcing to 0%.

To ensure that the critical heat shock of *C. glutamicum* is optimized for the AutoBioTech platform, a transformation experiment was performed at HSDs from 1 to 11 min. Additionally, three different plasmids were used with at least two replicates per combination of HSD and plasmid. Heat transfer at the reduced scale of 200 μL was expected to be similar to the standard scale of approximately 1000 μL.^30,43,44^ Thus, little deviation from the known optimal HSD of 6 min was expected.

**Figure 7:**
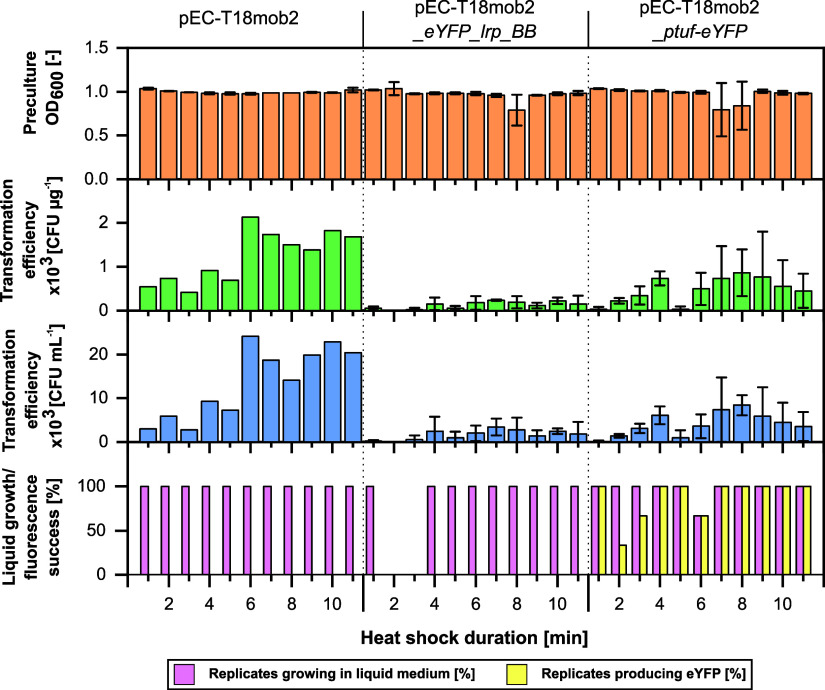
Automated electroporation of *C. glutamicum* at variable heat shock durations. Cell densities of *C. glutamicum* precultures. Transformation efficiency based on the amount of plasmid DNA. Transformation efficiency in CFUs relative to OD_600_ of competent cells. CFUs counted manually. Percentage of picked transformants with success of growth in liquid medium and production of eYFP. Error bars represent standard deviation of three replicates. As only two replicates were conducted with pEC-T18mob2 no standard deviation was calculated.

A monitoring of precultures to generate competent cells showed decreased growth in 3 samples, which could have originated from a technical deviation such as a lower culture volume (Figure 7). Nevertheless, all precultures were also used for inoculation of the main culture, which was expected to be homogeneous across all samples as little other deviations were observed between the precultures.

After electroporation at different HSDs, it was found that with plasmid pEC-T18mob2, the efficiency increased from, on average, 660 to 2123 CFU μg^–1^ when the HSD was 6 min compared to shorter durations. For HSDs longer than 6 min, a trend toward decreasing efficiencies was observed. With plasmids pEC-T18mob2_*eYFP_lrp_BB* and pEC-T18mob2_*ptuf-eYFP*, the efficiencies were much lower with less than 1000 CFU μg^–1^. Also due to high standard deviations, it was not possible to establish a clear trend between HSD and transformation efficiency for both plasmids. Therefore, in accordance with existing literature, a 6 min HSD was chosen as the standard for the AutoBioTech platform.^30^ Considering the transformation efficiency in relation to the OD_600_ of competent cells (CFU mL^–1^), similar trends were observed compared to CFU μg^–1^. Most importantly, the maximum electroporation efficiencies were much higher for all three plasmids compared to conjugation, e.g., 24 × 10^3^ CFU mL^–1^ versus 1 × 10^2^ CFU mL^–1^ for plasmid pEC-T18mob2, respectively. This indicates that automated electroporation may generally be more efficient than conjugation, which should however be tested for a diverse selection of plasmids.

After picking transformants and performing growth phenotyping, it was found that most of the picked colonies grew in liquid medium (Figure 7). Assuming that nongrowing colonies primarily originate from an inaccuracy in automated picking, they can be quantified in a mis-pick rate relative to the total number of picked colonies. Consequently, these colonies accounted for mis-pick rates of 0, 4.3, and 3.8%, which is within the expected range of 5.2% described in the Supporting Information (Figure S8). The low growth failure after electroporation confirmed an assumption that a biological effect during conjugation might affect the ability of transformants to grow in liquid medium (see Supporting Information). For the transformants with pEC-T18mob2_*ptuf-eYFP*, it was observed that not all replicates had produced eYFP at 2 and 3 min HSD and therefore likely had lost the *eYFP* gene. This indicates that the restriction system of *C. glutamicum* may not have been sufficiently inactivated at the short HSDs and restriction enzymes may have partially digested the plasmid in some transformants.

## Conclusions and Outlook

In this study, the AutoBioTech platform, which consists of a versatile combination of 14 devices, was developed together with workflows for the fully automated, high-throughput strain engineering of a Gram-negative and a Gram-positive microorganism. Automated transformation and growth screening was established for *E. coli*, and the observed variances in the final liquid cultures could be explained by cell viability effects during colony picking. Genetic modularity was introduced with the CIDAR MoClo system and extended for modular CRISPR/Cas9-based genome deletions. Specifically, the successful deletion of the *lacZ* gene was autonomously enabled by a combination of different gRNAs and homologous regions.

For *C. glutamicum*, two fully automated transformation procedures were established employing conjugation and electroporation, respectively. As a critical parameter for efficiency, the heat shock duration was adapted to the AutoBioTech platform and found to be sufficient for conjugation at 3 min (Figure S6), while no deviation from the standard 6 min was required for electroporation. Nevertheless, electroporation proved to be more efficient than conjugation by more than 1 order of magnitude.

The establishment of an automated electroporation workflow will not only affect strain construction capacities for *C. glutamicum*, but also make other industrially relevant Gram-positive organisms, such as *Bacillus subtilis*, rapidly accessible via the AutoBioTech platform. Potentially, this technique could even broaden the application spectrum of this platform to a diverse set of aerotolerant bacterial and eukaryotic organisms.

An overarching focus of further studies will also be to use the enormous amount of data that will be generated by the AutoBioTech platform for learning processes in the spirit of the design-build-test-learn cycle. In this way, parameter optimization will become a continuous process as the required data is generated during every workflow execution, regardless of the samples processed. Ultimately, the integration of machine learning could make strain engineering not only faster, but also more efficient and reliable than human decision-making.

## Material and Methods

### Strains, Plasmids and Growth Conditions

Organisms used in the automated genome engineering of *E. coli* were strains MG1655 and DH5α. Basic DNA parts (Level 0 plasmids) for MoClo were acquired from Addgene (Addgene, Cambridge, USA, Kit #1000000059) in glycerol stocks of transformed *E. coli* cells and isolated using the NucleoBond PC 100 Midi kit (Macherey-Nagel GmbH & Co KG, Düren, Germany). The specific plasmids used in this study are listed in Table S3 and all designed and assembled plasmids in this study are listed in Table S4. Organisms used in automated conjugation and electroporation workflows were *E. coli* S17–1 and *C. glutamicum* ATCC13032, also referred to as *C. glutamicum* wild type. Plasmids used for the transformation of *C. glutamicum* are listed in Table S7. pEC-T18mob2_*ptuf-eYFP* was created specifically for this study using standard molecular biology techniques employing *E. coli* DH5α.^45,46^

Unless specified otherwise, *E. coli* was grown in liquid Lysogeny Broth (LB) medium (Carl Roth GmbH + Co. KG, Karlsruhe, Germany) or on LB-agar plates with 15 g L^–1^ Agar–Agar (Merck, Darmstadt, Germany). *C. glutamicum* was grown in liquid BHI medium (Carl Roth GmbH + Co. KG, Karlsruhe, Germany), liquid BHI medium supplemented with 90 g L^–1^ sorbitol and 10 g L^–1^ glucose (BHISG) or BHISG-agar plates with 15 g L^–1^ Agar–Agar. Furthermore, *C. glutamicum* was grown on defined CGXII medium^47^ consisting of 20 g L^–1^ (NH4)_2_SO_4_, 1 g L^–1^ K_2_HPO_4_, 1 g L^–1^ KH_2_PO_4_, 5 g L^–1^ Urea, 42 g L^–1^ MOPS, 13.25 mg L^–1^ CaCl_2_ · 2 H_2_O, 0.25 g L^–1^ MgSO_4_ · 7 H_2_O, 0.01 g L^–1^ FeSO_4_ · 7 H_2_O, 0.01 g L^–1^ MnSO_4_ · H_2_O, 20 μg L^–1^ NiCl_2_ · 6 H_2_O, 0.313 mg L^–1^ CuSO_4_ · 5 H_2_O, 1 mg L^–1^ ZnSO_4_ · 7 H_2_O, 0.2 mg L^–1^ Biotin, 0.03 g L^–1^ protocatechuic acid and 20 g L^–1^d-glucose. All media were supplemented with 5 μg mL^–1^ tetracycline (Fluka BioChemica, Buchs, Switzerland), 50 μg mL^–1^ spectinomycin or 50 μg mL^–1^ kanamycin, depending on the expected resistance of the grown organism.

Cultivations of *E. coli* were carried out in an orbital shaking incubator at 37 or 30 °C and *C. glutamicum* was cultivated at 30 °C. Shake flask cultivations were shaken at 250 rpm with a 1:10 ratio of liquid to flask volume while MTP and deep well plate cultivations were shaken at 900 rpm with liquid volumes of 200 and 1200 μL, respectively. Additionally, automated cultivations were carried out at 90% relative humidity.

Cryo cultures were used as inocula for various cultivations in this study. They were created by cultivating a strain overnight in a shake flask, after which the OD_600_ was measured. The complete cultivation broth was transferred to a 50 mL falcon tube and centrifuged at 4000*g and 4 °C for 10 min. After that, the supernatant was decanted, and the cell pellet was resuspended in 15 mL of a 0.9% NaCl and 25% glycerol solution by pipetting up and down. The cell suspension was stored at −80 °C.

### Module I —DNA Assembly

For the Golden Gate assembly in Module I (Figure S1) of DNA parts, a master mix containing BsaI (R3733, NEB, Ipswich), T4 Ligase (M0202L, NEB, Ipswich) and T4 ligase buffer (B0202S, NEB, Ipswich) was prepared. The final concentrations per reaction were 1, 10 U μL^–1^ and 1 times concentration, respectively. In the case of automated MoClo reactions, 70 ng μL^–1^ of the plasmids containing the destination vector, terminator and gene of interest were added to each reaction. Other DNA parts such as plasmids containing promoters and ribosome binding sites were also added at 70 ng μL^–1^. The reactions were prepared in a PCR plate and filled to a total volume of 20 μL using nuclease-free water (QIAGEN GmbH, Hilden, Germany). After sealing the PCR plate, the automated thermal cycler ATC applied 25 cycles of 37 °C for 1.5 min and 16 °C for 3 min. Finally, the temperature was adjusted to 50 °C for 5 min and subsequently to 80 °C for 10 min. The PCR plate was stored at 4 °C until further use.

### Module II—Chemically Competent Cells

To produce chemically competent *E. coli* cells, a preculture was conducted in 25 mL medium in a 100 mL shake flask that was incubated at 250 rpm and 37 °C for 16 h. In a square well deep well plate, 1200 μL LB-medium per well were inoculated with 40 μL preculture each and cultivated at 900 rpm and 30 °C for 4 h. Cells were washed by centrifuging at 3500 rpm for 5 min and discarding 1200 μL supernatant. The pellet was resuspended in 800 μL 0.1 M CaCl_2_ by pipetting 400 μL up and down 10 times. The deep well plate was incubated on a cooling carrier set to 2 °C for 20 min, after which the deep well plate was centrifuged again at 3500 rpm for 5 min and 800 μL supernatant was discarded. Next, the pellet was resuspended in 50 μL 0.1 M CaCl_2_ and 15% glycerol by pipetting 40 μL up and down 15 times. Finally, the cell suspension was transferred to a PCR plate, sealed and stored at 4 °C for up to 24 h until use.

### Module III—Heat Shock Transformation

To transform chemically competent *E. coli* cells with assembled DNA, 10 μL DNA suspension (Module I) were added to 50 μL cell suspension (Module II), after which 40 μL of the mixture were pipetted up and down 10 times. The PCR plate containing the mixture was incubated on a cooling carrier set to 4 °C for 30 min and consequently sealed. A heat shock was conducted in a thermal cycler at 42 °C for 45 s and subsequently the PCR plate was cooled to 4 °C for 5 min. Next, 80 μL SOC-medium were added to each sample and 140 μL from each sample were transferred to a V-well-plate already containing a further 110 μL of SOC-medium. Transformant recovery was conducted by incubating the V-well-plate at 900 rpm and 37 °C for 45 min. After that, the V-well plate was centrifuged at 3500 rpm for 5 min, 200 μL supernatant were removed and the pellet was resuspended in the remaining medium by pipetting 50 μL up and down 10 times. 3 μL of each sample were spotted onto two LB-agar plates containing the appropriate selection antibiotic, 20 μg mL^–1^ 5-bromo-4-chloro-3-indolyl ß-D-galactopyranoside (X-GAL, Carl Roth GmbH+Co. KG, Karlsruhe, Germany) and, when applicable, 0.1 mM isopropyl-ß-D-thiogalactopyranoside (IPTG, Sigma-Aldrich, Taufkirchen, Germany). Finally, the agar plates were incubated for 24 h at 30 °C and stored at 4 °C until further processing.

### Module IV—Picking and Screening

Colonies from module III were analyzed for coloring (blue/white) by the Pickolo software and picked into a MTP containing 200 μL LB-medium with the appropriate selection antibiotic per well. The MTP was incubated at 900 rpm and 30 °C for 18 h during which the OD_600_ was measured once per hour. For GFP-producing transformants, fluorescence intensity was measured at λ_ex_: 479 ± 20 nm and λ_em_: 520 ± 20 nm.

### Phenotyping via Mini Pilot Plant Technology

For the phenotyping of *E. coli* transformants via Mini Pilot Plant technology, the precultures were cultivated in LB medium with the appropriate selection antibiotic in a MTP sealed with a gas-permeable membrane. The plate was shaken at 1000 rpm for 18 h, utilizing a benchtop device within a temperature-controlled cabinet (Edmund Bühler H5, Hechingen, Germany). After that, the preculture OD_600_ was measured and set to 0.1 by dilution with LB-medium. 50 μL of the OD-normalized precultures were utilized to inoculate main cultures in 48-well flower plates, which were sealed with gas-permeable membranes and cultivated in a BioLector Pro (m2p-labs, now part of Beckman Coulter Life Sciences, Baesweiler, Germany). The main culture was cultivated for 24 h at 37 °C and 1000 rpm in 1000 μL LB medium with a humidity of 85%. GFP fluorescence was measured online by the BioLector Pro (GFP Filter ID204, λ_ex_: 488 nm, λ_em_: 520 nm).

### Plasmid Construction for CRISPR/Cas9-Based Genome Editing

Plasmid pEdit_AE was constructed by standard molecular biology techniques based on plasmids pTarget^16^ and DVK_AE^13^ using restriction enzymes NheI and *Bgl*II. Specifically, the lacZ fragment from DVK_AE was amplified using primers P3 and P4 (see Table S6) and cloned into pTarget. The homologous regions used for genome editing were amplified from the *E. coli* BL21 genome, using primers P5 and P6 (see Table S6).

### *E. coli* Genome Editing

*E. coli* MG1655 was transformed with plasmids pCas and pEdit via modules II and III. Instead of direct screening, transformants were picked into an MTP with 200 μL liquid LB-medium supplemented with 10 mM l-arabinose, 50 μg mL^–1^ kanamycin and 50 μg mL^–1^ spectinomycin per well. The plate was incubated at 30 °C and 1000 rpm for 6 h, after which the cultivation broth was diluted by factors 1:8, 1:64, 1:512, and 1:4096. These dilutions were plated on LB-agar plates containing kanamycin and spectinomycin and incubated analogue to module III.

### Plasmid Curing

Curing of pEdit was conducted by picking colonies from genome editing into a V-well plate with 200 μL LB-medium supplemented with kanamycin and 0.1 mM IPTG per well. The V-well plate was incubated at 30 °C and 1000 rpm for 6 h and subsequently a 1:100 dilution of the culture broth was plated on LB-agar plates supplemented with kanamycin and IPTG. Again, the plates were cultivated analogue to module III and subsequent curing of pCas could be conducted by repeating the pEdit curing process without media supplementation and at an incubation temperature of 37 °C. For the last plating step, the culture broth was diluted by factors 1:8, 1:64, 1:512, and 1:4096 and all dilutions were plated on an LB-agar plate without supplementation.

### *E. coli* Genome Editing Phenotyping

Edited clones were phenotyped by picking colonies into a MTP containing 200 μL LB-medium with the appropriate selection antibiotic and 20 μg mL^–1^ X-Gal per well. The MTP was cultivated at 1000 rpm and 30 °C for 18 h, after which unsuccessful editing could be determined by blue coloration of the cultivation medium.

### Conjugation and Electroporation of *C. glutamicum*

Existing conjugation^23^ and electroporation^43^ protocols of *C. glutamicum* were adapted and optimized for compatibility with automated devices (see Supporting Information for details on conjugation). Prior to electroporation of *C. glutamicum*, competent cells were generated according to a protocol adopted from Jiang et al., 2017.^43^ For this purpose, three consecutive cultivations were performed: The first preculture consisted of 200 μL of BHI medium in each well of an MTP inoculated with 5 μL of a *C. glutamicum* cryoculture each. After incubation for at least 4 h and up to 12 h at 30 °C and 900 rpm, the first preculture was used as an inoculum for the second preculture. 200 μL of BHISG medium in each well of an MTP was inoculated with 5 μL of the first preculture. The second preculture was again incubated for 4 h at 30 °C and 900 rpm. Subsequently, the main culture was conducted in a DWP with 1 mL BHISG medium supplemented with 4 g L^–1^ glycine and 0.1% (v v^–1^) Tween80 per well. 5 μL of the second preculture was used to inoculate each well of the main culture. After incubation for 5 h at 30 °C and 900 rpm, the main culture was stored at 4 °C for 20 min without shaking. The cells of the main culture were washed in the following steps: Centrifugation at 4500 rpm and 4 °C for 5 min, after which 820 μL supernatant was discarded. 900 μL of 10% (w v^–1^) glycerol was added to each well and the pellet was resuspended by pipetting 450 μL up and down 20 times. The cells were then washed again, this time discarding 900 μL supernatant after centrifugation. The plate was then centrifuged a third time and 900 μL of supernatant was discarded. The pellet was resuspended in the remaining liquid by pipetting 60 μL up and down 20 times. Finally, the competent cells were either stored at 4 °C for up to 1 h or used fresh.

For electroporation, 20 μL of the previously generated competent *C. glutamicum* cells were transferred to each well of a 96-well Nucleocuvette Plate (Lonza Cologne GmbH, Cologne Germany). 3 μL of plasmid solution with a concentration of 20–50 ng μL^–1^ was added to each well. The electroporation plate was then placed in the 4D-Nucleofector 96-well Unit (Lonza Cologne GmbH, Cologne Germany) and the 4D-Nucleofector Core Unit (Lonza Cologne GmbH, Cologne Germany) was used to apply the bacterial pulse F to each well. Immediately after electroporation, 180 μL of BHISG medium prewarmed to 46 °C was added to each well. As much liquid as possible was transferred from the electroporation plate to a PCR plate and heat shocked at 46 °C for 6 min in a thermal cycler. From the PCR plate, each well was transferred to the corresponding well in a VWP. This VWP was incubated at 30 °C for 1–2 h without shaking. After this recovery time, 5 μL of each well was spotted onto a BHISG agar plate in SBS-format containing the appropriate selection antibiotic. The VWP was then centrifuged at 4500 rpm and 20 °C for 5 min. 120 μL of the supernatant per well was discarded and the pellet was resuspended in the remaining liquid by pipetting 25 μL up and down 20 times. Subsequently, 5 μL per well of the concentrated cell suspension was spotted onto a second BHISG agar plate containing the appropriate selection antibiotic. Both agar plates were left open to dry for 10 min and then incubated at 30 °C for 40 h with a lid. The agar plates were then stored at 4 °C until further use.

Transformation efficiencies were calculated first in CFU mL^–1^, allowing for comparisons between conjugation and electroporation, and second in the standard unit CFU μg_DNA_^–1^ for electroporation (see Supporting Information for details on the calculations).

After transformations, a growth test and, if applicable, fluorescence phenotyping were conducted by two successive cultivations. Colonies were picked into and cultivated in BHI medium in an MTP with the appropriate selection antibiotic. Also, 50 μg mL^–1^ nalidixine were added to select against *E. coli*.^48,49^ The MTP was incubated for 20 h with start- and end-point measurements of the OD_600_ in a microplate reader. After that, 5 μL of the cultures were used as an inoculum for a second culture in an MTP with CGXII medium and the appropriate selection antibiotic. Again, the MTP was incubated for 20 h with start- and end-point measurements of the OD_600_ in a microplate reader. Additionally, when eYFP was produced, the fluorescence intensity was measured at λ_ex_: 488 ± 20 nm and λ_em_: 525 ± 20 nm. The fluorescence intensity was divided by the OD_600_ to receive biomass-specific fluorescence intensity values.

## Supporting Information Available

Supporting Information S1 contains additional results and discussion as well as additional material and methods (PDF)
