# Supplementary material for: AutoBioTech—A Versatile Biofoundry for Automated Strain Engineering
Source: ACS Synth Biol. 2024 Jul 8;13(7):2227–37. doi: 10.1021/acssynbio.4c00298 (PMC11264319; doi:10.1021/acssynbio.4c00298)
Supplement: Supplementary file 1 — sb4c00298_si_001.pdf [file sb4c00298_si_001.pdf]

Supporting Information

AutoBioTech – A Versatile Biofoundry for Automated Strain Engineering

Tobias Michael Rosch [†,#], Julia Tenhaef [†,#], Tim Stoltmann [†,#], Till Redeker [†], Dominic Kösters [†,‡], Niels Hollmann [†,‡], Karin Krumbach [†], Wolfgang Wiechert [†], Michael Bott [†,§], Susana Matamouros [†], Jan Marienhagen [†,‡], and Stephan Noack [*,†]

[†] Institute of Bio- and Geosciences, IBG-1: Biotechnology, Forschungszentrum Jülich, D-52425 Jülich, Germany

[‡] Institute of Biotechnology, RWTH Aachen University, Worringer Weg 3, D-52074 Aachen, Germany

[§] The Bioeconomy Science Center (BioSC), Forschungszentrum Jülich, D-52425 Jülich, Germany

[#] These authors contributed equally

* Corresponding author:
E-mail: s.noack@fz-juelich.de
Tel.: +49 2461 61-6044

Content

Additional results and discussion **3**

Basic workflow for plasmid-based strain library construction 3

Two-species population model for *E. coli* 5

MoClo-compatible CRISPR/Cas system for *E. coli* 7

Fully automated conjugation of *C. glutamicum* and effect of heat shock duration on transformation efficiency 7

Automated electroporation of *C. glutamicum* 12

Additional material and methods **14**

Devices included in the AutoBioTech platform 14

Automated conjugation of *Corynebacterium glutamicum* 14

Calculation of transformation efficiency 15

References **20**

Additional results and discussion

Basic workflow for plasmid-based strain library construction

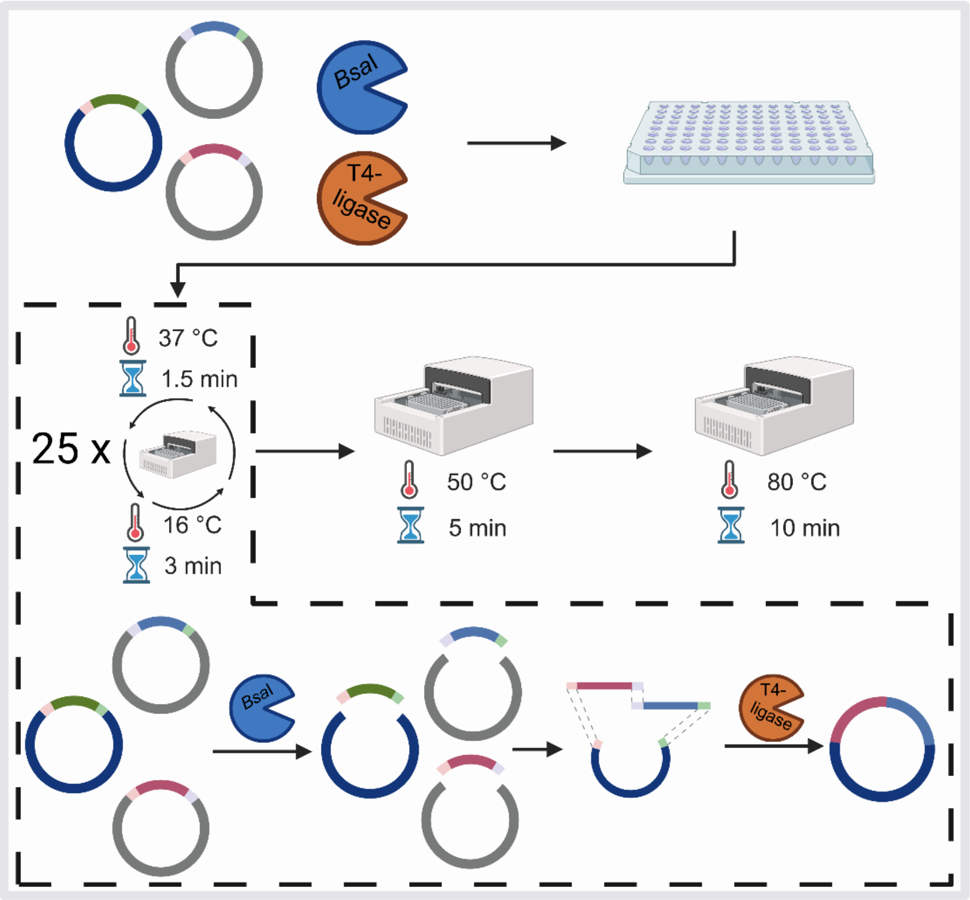

Figure S1: Module I – DNA assembly. Mixing of vectors containing DNA parts with restriction enzyme BsaI and T4-DNA ligase. Thermocycler protocol with cycles and final temperature steps. Visualization of DNA assembly via excision of DNA parts and ligation in the destination vector. Created with BioRender.com

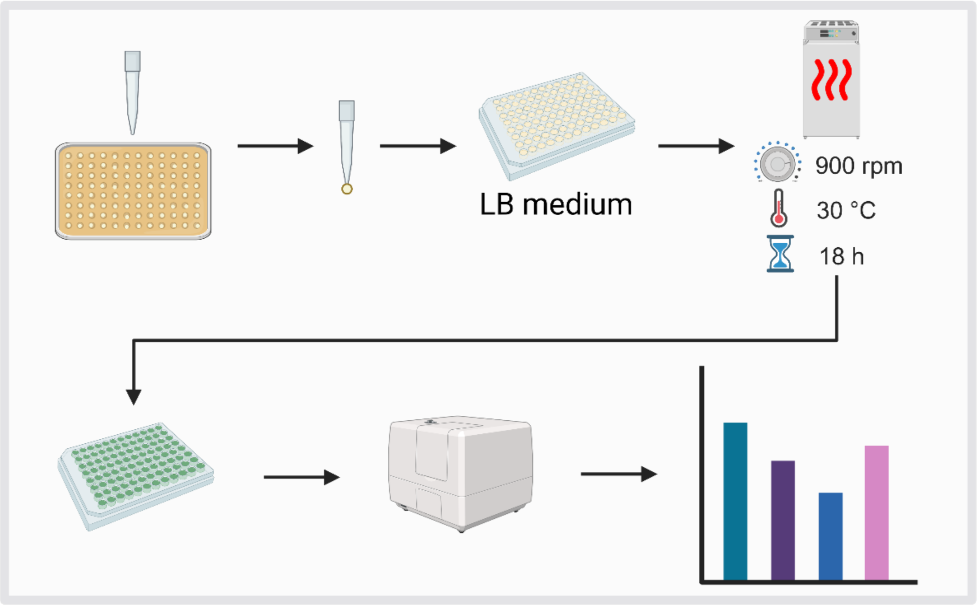

Figure S2: Module IV – Colony picking, incubation and screening. Picking of transformant colonies into microtiterplate with LB medium and incubation. Measurement of OD_{600} and further spectrophotometric values in the Synergy H1 plate reader. Created with BioRender.com

Two-species population model for *E. coli*

For the analysis of growth phenotypes from the application of the basic cloning workflow for *E. coli* the following two-species population model was formulated:

$$X_{tot} = X_v + X_{nv} \qquad X_{tot}(t_0) = X_{tot,0} \tag{1}$$

$$X_{nv} = X_{tot,0} \cdot (1 - f) \qquad f \in [0,1] \tag{2}$$

$$\frac{dc_{X_v}}{dt} = \dot{X}_v = \mu(S) \cdot X_v \qquad X_v(t_0) = X_{tot,0} \cdot f \tag{3}$$

$$\frac{dc_S}{dt} = \dot{S} = q_S \cdot X_v \qquad S(t_0) = S_0 \tag{4}$$

with

$$\mu(S) = \mu_{max} \frac{S}{K_S + S} \tag{5}$$

$$q_S = -\frac{1}{Y_{X/S}} \cdot \mu(S) \tag{6}$$

Here X_{tot}, X_v and X_{nv} denote the total, viable and non-viable biomass concentration, respectively. The latter accounts for potentially inactive cells in the initial biomass $X_{tot,0}$ after transfer from solid to liquid medium. Both are linked via the fraction f, which was introduced as a replicate-dependent (local) parameter. The specific growth rate of viable biomass $\mu(S)$ is dependent on one limiting carbon source S, which is modelled by classical Monod kinetics with strain-specific (global) parameters μ_{max} and K_S. The substrate consumption rate q_S is related to biomass growth by the yield coefficient $Y_{X/S}$, which was fixed to 0.5 g g^{-1} [1].

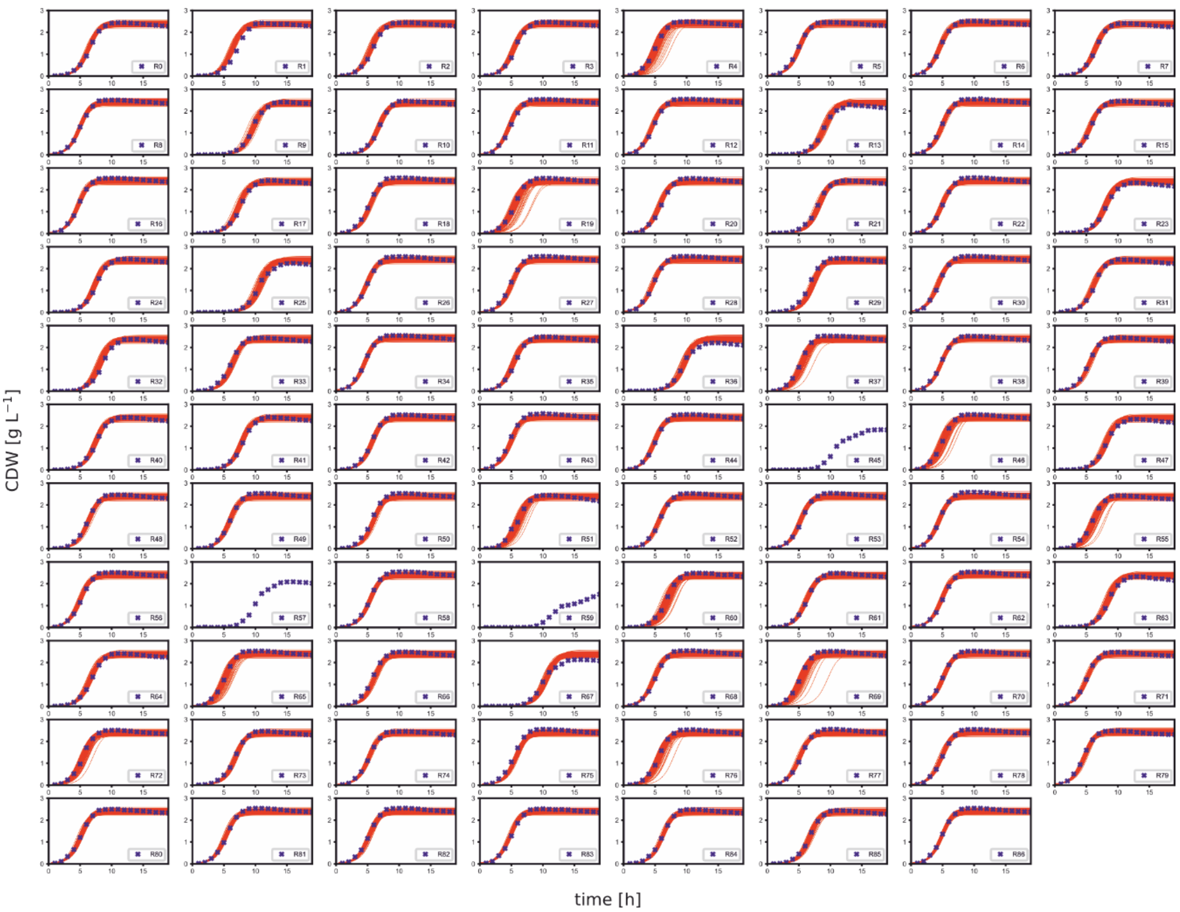

Figure S3: Measurements of cell dry weight (blue crosses) and model fits (red straight lines) from the 87 liquid cultures of *E. coli* transformants. After picking, three colonies showed significantly altered growth profiles (R45, R57, R59). These were excluded from model-based analysis.

MoClo-compatible CRISPR/Cas system for *E. coli*

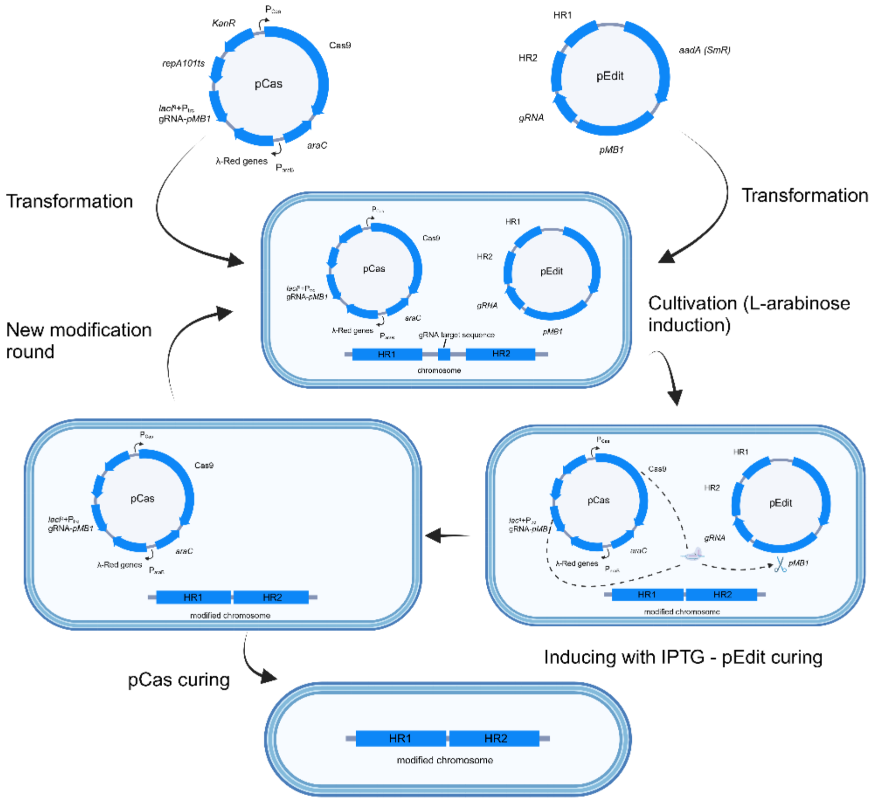

Figure S4: Schematic of the MoClo-compatible CRISPR/Cas system. Transformation of pCas and the assembled pEdit into *E. coli*; for pEdit assembly see Figure 5A. Genome editing by Cas9, gRNA and L-arabinose induced λ-Red recombinase production. Curing of pEdit by IPTG induction of pEdit-specific gRNA transcription. Curing of pCas by incubation at 37 °C. Figure adapted from Jiang et al., 2015 [2]. Created with BioRender.com.

Fully automated conjugation of *C. glutamicum* and effect of heat shock duration on transformation efficiency

The conjugation of *C. glutamicum* was adapted from our previous workflow [3] and further optimized as a scaled-down version to the fully automated laboratory environment of AutoBiotech. Especially large volumetric-scale changes by more than one order of magnitude require the adaption of process parameters due to changes in mass and energy transfer characteristics [4, 5].

First, the cultivation of *C. glutamicum* prior to conjugation was now performed in a 96-well MTP instead of a shake flask. Second, the colony picking from agar plates was automated using the Pickolo device integrated into the AutoBioTech platform. Third, a PCR plate in conjunction with a thermal cycler was used to mimic the 9 min heat shock previously performed with a shake flask in a water bath. As a result, a fully automated workflow for the conjugation of *C. glutamicum* could be established (Figure S5), which is an equivalent to Module III for *E. coli* (Figure 2B).

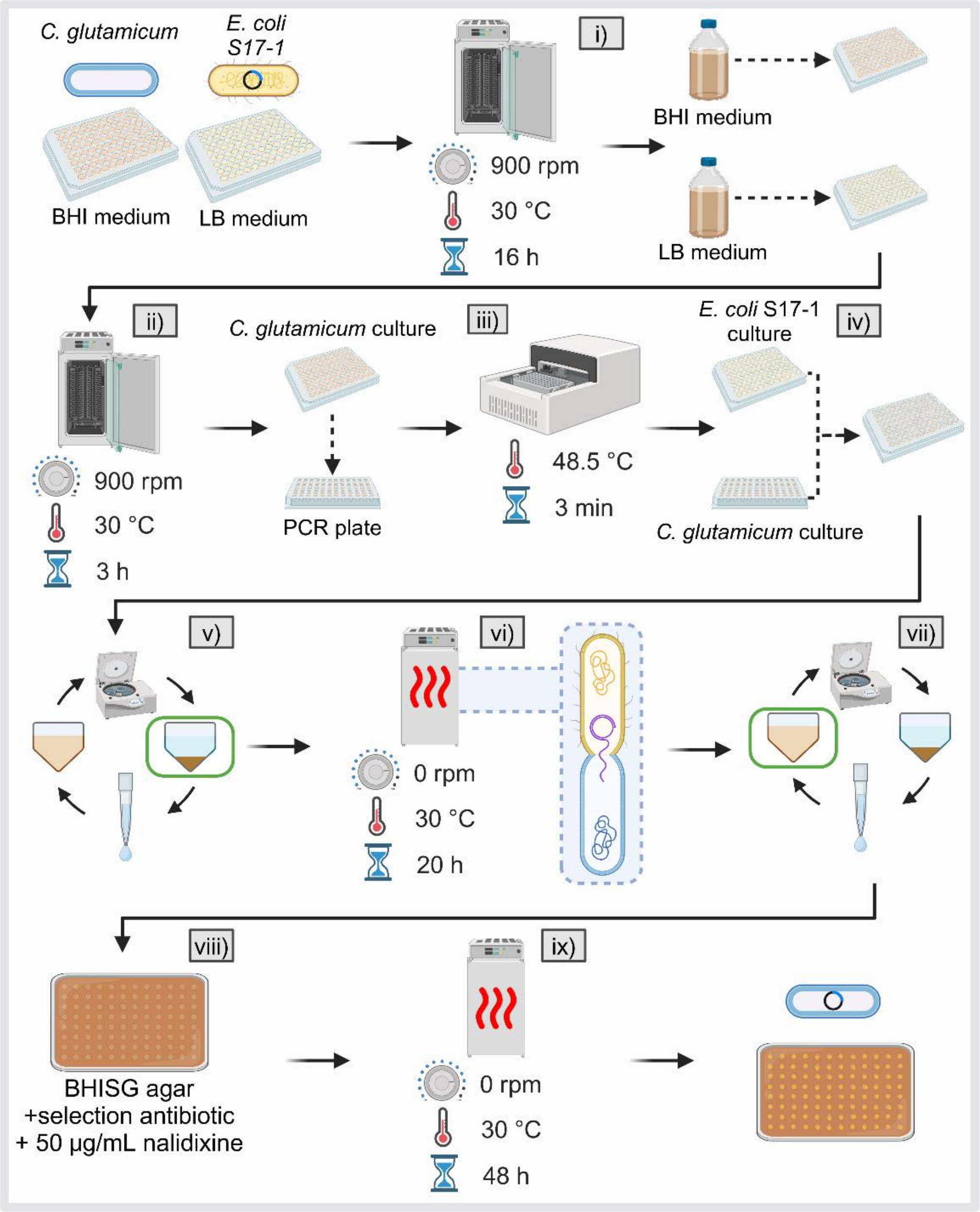

Figure S5: Workflow of automated conjugation of *C. glutamicum*. i) precultures for *E. coli*-donor and *C. glutamicum*-acceptor cells, ii) main cultures, iii) heat shock of *C. glutamicum*, iv) culture combination v) cell washing, vi) conjugation, vii) pellet resuspension, viii) plating and ix) agar incubation. Detailed descriptions can be found in the additional material and methods section. Continuous arrows represent process flow; dashed arrows represent liquid transfer. Created with BioRender.com.

The effect of scale changes in thermal processes is not always predictable, as the exact parameters of thermal devices are rarely specified in their data sheets. Therefore, the optimal heat shock duration (HSD) prior to conjugation was investigated. Preliminary tests showed that a water bath required 7 min to heat culture broth to the set point temperature of 48.5 °C [3], while the thermal cycler in the AutoBioTech platform reached the same temperature in less than 1 min. Therefore, it was expected that a HSD of 9 min in a thermal cycler could have adverse effects.

To test this, *C. glutamicum* was transformed using the automated conjugation procedure in which the HSD was varied between 1 – 10 min. In addition, three conjugatable plasmids pEC-T18mob2, pEC-T18mob2*_eYFP_lrp_BB* and pEC-T18mob2*_ptuf-eYFP* were used to identify potential effects related to gene expression (Table S7). In conjugation experiments, the employed plasmid mass is unknown because it is present exclusively in *E. coli* S17-1 donor strain. Previously, it was found that the density of the *C. glutamicum* culture has the greatest impact on efficiency [6]. Therefore, transformation efficiency was calculated as colony forming units (CFU) versus optical density (OD) at 600 nm (OD_{600}) of the *C. glutamicum* culture according to equation 8 (see additional material and methods).

During the conjugation procedure, growth of *C. glutamicum* and *E. coli* S17-1 cultures was analysed by OD_{600} measurement. It was found that *C. glutamicum* and *E. coli* S17-1 cultures grew to almost homogenous final OD_{600}, respectively. Transformations with pEC-T18mob2 displayed a sharp increase in efficiency when the HSD exceeded 1 min, up to 103 CFU mL^{-1} at 3 min, and a decrease as it exceeded 5 min (Figure S6). Transformations with pEC-T18mob2*_eYFP_lrp_BB* resulted in a similar trend, with an efficiency maximum of 96 ± 22 CFU mL^{-1} at 3 min. Prolonged HSDs over 5 min again resulted in a decreased transformation efficiency of approximately 33 CFU mL^{-1}, which may have been the result of lower cell viability at prolonged heat exposure.

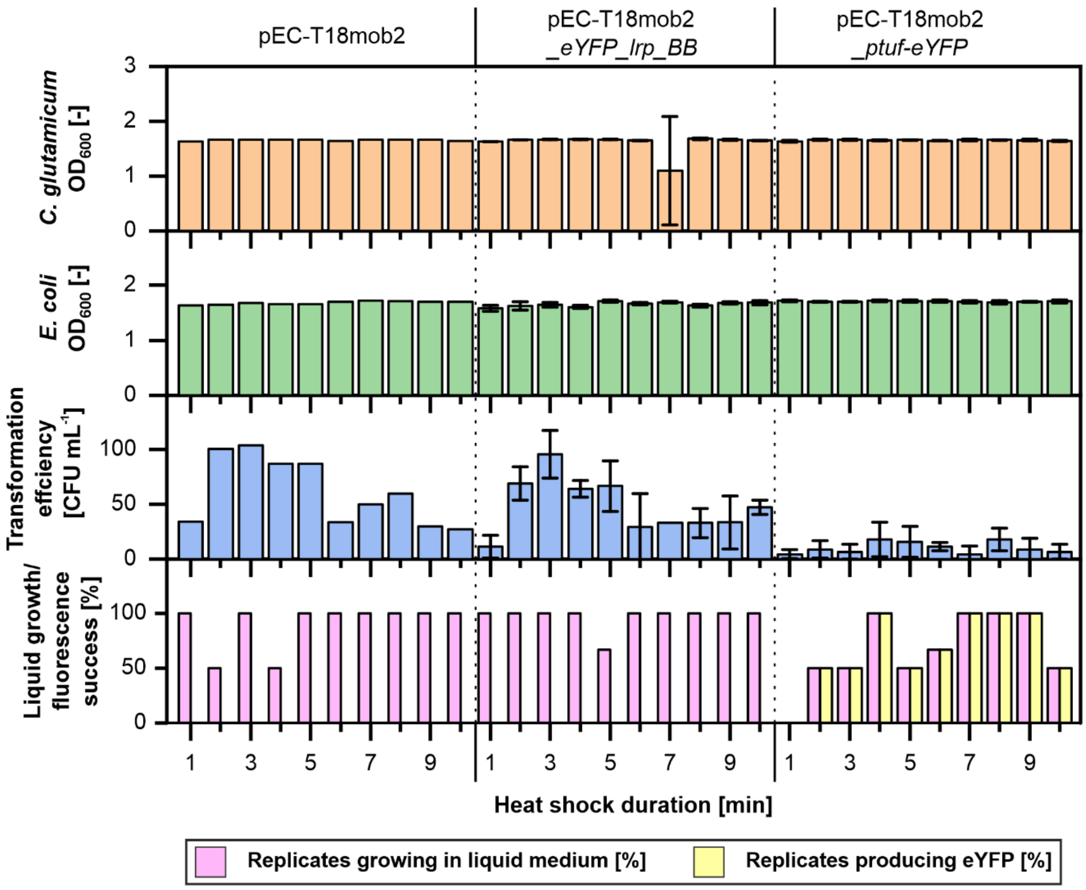

Figure S6: Automated conjugation of *C. glutamicum* at variable heat shock durations. Cell densities of *C. glutamicum* and *E. coli* S17-1 cultures. Transformation efficiency – CFUs counted manually. Percentage of picked transformants with successful growth in liquid medium and production of eYFP. Mean values were estimated from two (pEC-T18mob2) and three (pEC-T18mob2*_eYFP_lrp_BB* and pEC-T18mob2*_ptuf-eYFP*) replicate transformations, respectively. Error bars represent standard deviation of three replicates. As only two replicates were conducted with pEC-T18mob2 no standard deviation was calculated.

This result confirmed the assumption that the original HSD of 9 min is too long at the reduced scale. With pEC-T18mob2_*ptuf-eYFP*, the transformation efficiency was on average 75 % lower than with pEC-T18mob2_*eYFP_lrp_BB* and the relatively large error observed for each of the conditions tested rendered the results inconclusive. In preliminary tests, it was found that *E. coli* S17-1 grows significantly slower with plasmid pEC-T18mob2_*ptuf-eYFP* than with the other two tested plasmids (Figure S7). Possibly, the constitutive production of enhanced yellow fluorescent protein (eYFP) und control of the promotor *ptuf* has an adverse effect on conjugation through interaction with the protein machinery for conjugative DNA transfer or due to metabolic burden effects. Nonetheless, a HSD of 3 min was concluded the optimal duration in this experimental setup.

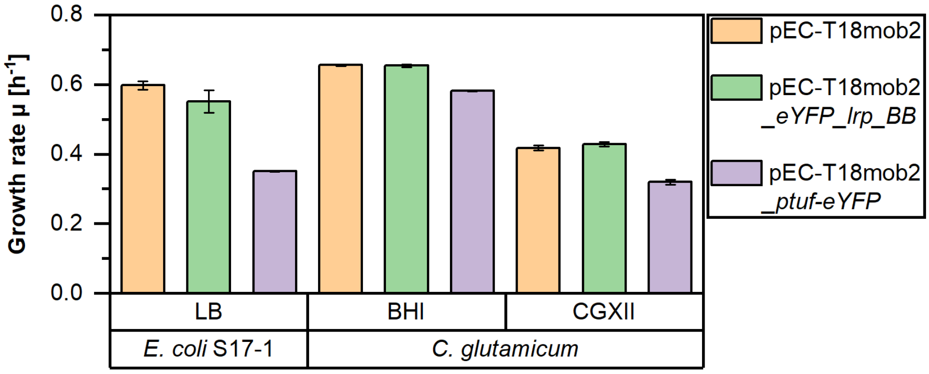

Figure S7: Growth rates of *E. coli* S17-1 and *C. glutamicum* with three different plasmids. *E. coli* S17-1 cultivated in LB medium, *C. glutamicum* cultivated in BHI and CGXII medium. At least 3 h of exponential growth, 4 replicates per data point. Orange: pEC-T18mob2, Green: pEC-T18mob2_*eYFP_lrp_BB* and Purple: pEC-T18mob2_*ptuf-eYFP*.

While colony formation is a suitable indicator for transformation efficiency, it does not reflect whether colonies are viable to grow in liquid medium[3]. Therefore a growth test and fluorescence phenotyping were performed with one colony from each replicate transformation by successive cultivation in liquid Brain-Heart-Infusion (BHI) and CGXII medium. In a 77 replicate picking experiment of *C. glutamicum* wild type colonies, the lag phase after colony picking was observed to reach up to 16 h (Figure S8).

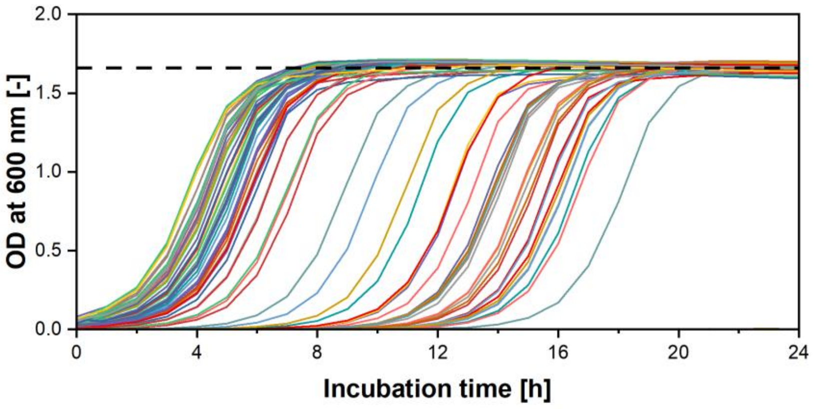

Figure S8: Cultivation of *C. glutamicum* WT in liquid media following automated colony picking. Using the Pickolo colony picker (SciRobotics, Kfar Saba, Israel). 77 colonies were picked into separate wells of standard clear, flat microtiter plate filled with liquid BHI medium. Cultivation was performed in a 200 µL BHI medium in a flat transparent MTP at 30 °C and 900 rpm. 4 colonies, corresponding to 5.2 %, did not grow or were missed during picking.

Consequently, replicates without significant growth in BHI medium were not disregarded but still used as an inoculum for CGXII medium. If growth occurred in CGXII medium, this was regarded a viable colony. Fluorescence data was also recorded for transformants with pEC-T18mob2_*ptuf*-*eYFP* as the plasmid leads to constitutive eYFP production, and resulting data confirmed plasmid integrity in all picked agar colonies. Unexpectedly, for transformants with the biosensor plasmid pEC-T18mob2_*eYFP_lrp_BB*, no difference in eYFP fluorescence intensity could be measured compared to transformants with pEC-T18mob2. This was true even under the induction with alanine−leucine dipeptide (data not shown), and is not in accordance with our previous work [3]. Therefore, the biosensor encoded on plasmid pEC-T18mob2_*eYFP_lrp_BB* was found to be dysfunctional so that no fluorescence data could be recorded for transformants harbouring this plasmid (Figure S6).

For each plasmid, colonies were picked that did not grow in liquid medium. However, only one colony was picked from each replicate transformation, so it was not possible to clearly distinguish an inaccuracy in automated picking from a biological reason for cells not growing in liquid media. Therefore, assuming that only picking inaccuracies were observed, "mis-pick" rates of 10.0 %, 3.4 %, and 33.4 % were estimated for transformants with plasmids pEC-T18mob2, pEC-T18mob2_*eYFP_lrp_BB* and pEC-T18mob2_*ptuf*-*eYFP*, respectively.

In the separate experiment with 77 pickings, an error rate of 5.2 % was determined for the AutoBioTech platform (Figure S8). On one hand, this suggests that non-growing colonies for transformants with pEC-T18mob2 and pEC-T18mob2_*eYFP_lrp_BB* are likely due to picking inaccuracies. On the other hand, colonies with pEC-T18mob2_*ptuf*-*eYFP* likely failed to grow in time due to a slower growth rate (see Figure S7) or a biological reason, which might be related to the poorer transformation efficiency with this plasmid.

Automated electroporation of *C. glutamicum*

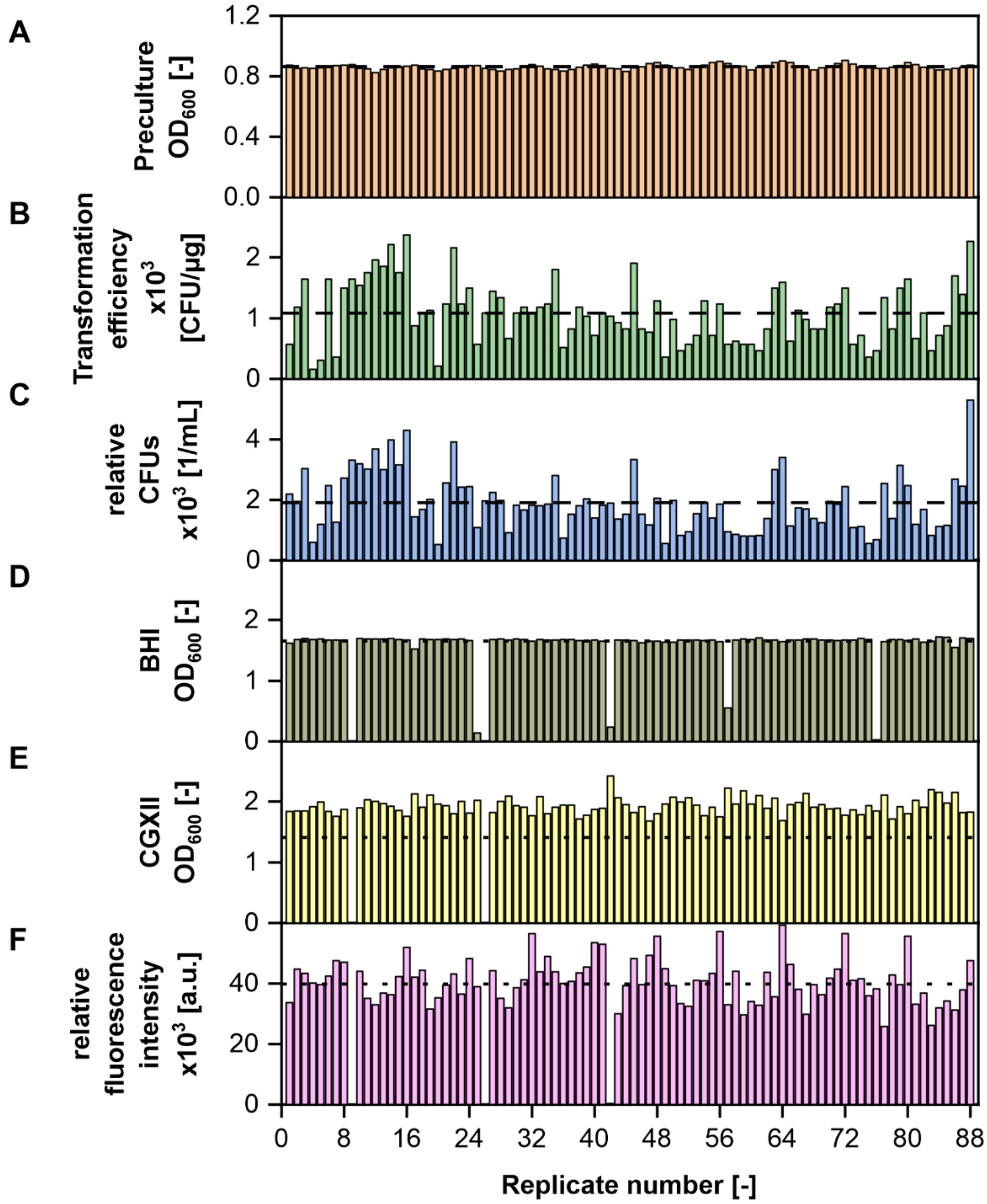

Figure S9: Semi-automated electroporation of *C. glutamicum* with plasmid pEC-T18mob2_*ptuf eYFP* in 88 replicates. (**A)** Cell density of *C. glutamicum* preculture. (**B)** Transformation efficiency in CFUs per mass of plasmid DNA x 10^3. (**C)** Transformation efficiency in CFUs relative to OD_{600} of competent cells - OD_{600} overestimated to 10 as measurement was unavailable. CFUs counted manually (**D)** Cell density of the BHI culture of picked transformants. (**E)** Cell density of the CGXII culture of picked transformants. (**F)** Biomass-specific relative fluorescence intensity (excitation: 488 nm; emission: 525 nm) x 10^3 of picked transformants. Horizontal dashed lines represent mean of all replicates (**A**-**C**). Horizontal dotted lines represent positive controls **(D-F)**.

At the molecular level, electroporation causes increased permeability of the cell membrane through the formation of pores [7, 8]. Based on mechanistic models, it is expected that mass transfer across the membrane is inversely proportional to the size of molecules passing through it [9]. Therefore, when using electroporation to introduce plasmid DNA into cells, transformation efficiency decreases with increasing plasmid size, which has been observed by several authors, especially for plasmid sizes larger than 4,000 bp [10-12].

The same effect was also observed in this study (Table S1). The smallest plasmid pEC-T18mob2 caused the highest transformation efficiency of 9.14 x 10^{14} CFU mol^{-1}, whereas the 903 bp larger plasmid pEC-T18mob2_ptuf-eYFP resulted in an efficiency of only 2.42 ± 1.72 x 10^{14} CFU mol^{-1}. These results suggest that plasmid size still has a noticeable effect on electroporation efficiency at the miniaturized scale of the 4D-Nucleofector®, so the amount of DNA added for each plasmid needs to be adjusted to achieve an optimal trade-off between material consumption and robustness of the protocol. Future studies could focus on creating a model to predict the optimal plasmid mass as a function of plasmid size and possibly the optical density of competent cells.

Table S1: **Effect of plasmid size on electroporation efficiency.**

Maximum molar transformation efficiency observed in automated electroporation experiments with *C. glutamicum*.

**Plasmid**	**Size [bp]**	**Molecular Mass x 10^6 [Da]**	**Maximum transformation efficiency x 10^{14} [CFU mol^{-1}]**
pEC-T18mob2	6209	3.84	9.14
pEC-T18mob2_*ptuf-eYFP*	7112	4.39	2.42 ± 1.72
pEC-T18mob2_*eYFP_lrp_BB*	7378	4.56	0.97 ± 0.97

Additional material and methods

Devices included in the AutoBioTech platform

Table S2: Devices included in the AutoBioTech platform.

Device type	Device name	Manufacturer	Registered seat
Robotic manipulator arm	Spinnaker Microplate Robot	Thermo Scientific™	Waltham, USA
Liquid handler	Fluent	Tecan Austria GmbH	Grödig, Austria
Static incubator	Cytomat 10 C450	Thermo Scientific™	Waltham, USA
Shaking incubator	Cytomat 2 C450-LiN	Thermo Scientific™	Waltham, USA
Centrifuge	Rotanta 460 robotic	Andreas Hettich GmbH & Co. KG	Tuttlingen, Germany
Heated/cooled shaker	BioShake D30-T elm	QINSTRUMENTS GmbH	Jena, Germany
Positive pressure unit	Resolvex® M10	Tecan Austria GmbH	Grödig, Austria
Colony picker	Pickolo	SciRobotics	Kfar Saba, Israel
Photospectrometric Plate Reader	Synergy H1	Agilent Technologies	Santa Clara USA
Thermal cycler	Automated Thermal Cycler (ATC) System	Applied Biosystems - Thermo Scientific™	Waltham, USA
Electroporator	4D-Nucleofector® Core Unit and 96-well Unit	Lonza	Basel, Switzerland
Fridge/Freezer	STR44	LiCONiC AG	Mauren, Liechtenstein
Plate peeler	XPeel®	Brooks Life Sciences	Chelmsford, Massachusetts, USA
Plate sealer	ALPS5000	Thermo Scientific™	Waltham, USA

Automated conjugation of *Corynebacterium glutamicum*

The protocol for the automated conjugation of *C. glutamicum* was adapted from Tenhaef et al., 2021 [3]. First, precultures of *E. coli* S17-1 and *C. glutamicum* were performed in MTPs with 80 µL of LB medium containing the appropriate selection antibiotic and 100 µL of BHI medium per well, respectively. Each well was inoculated with 5 µL of a cryoculture of the respective organism and the OD_{600} in each well was measured using a microplate reader. The plates were incubated at 30 °C and 900 rpm for 16 h, after which the OD_{600} was measured again. Each well was filled with fresh medium to a total volume of 200 µL and the OD_{600} was measured. The main culture was started by incubating the plates at 30 °C and 900 rpm. After 3 h, this cultivation was terminated and the final OD_{600} was measured. The entire *C. glutamicum*

cultivation volume from each well was transferred to the corresponding well in a PCR plate. The PCR plate was transferred to a thermal cycler and heat shocked at 48.5 °C for 3 min.

In a V-well plate (VWP), 50 µL of the *E. coli* S17-1 culture and 150 µL of the *C. glutamicum* culture were combined. The VWP was centrifuged at 4500 rpm and 20 °C for 5 min. 140 µL of the supernatant was discarded and the pellet was resuspended in 200 µL fresh LB medium without antibiotics by shaking the plate at 1000 rpm for 2 min. The centrifugation was repeated and 160 µL of the supernatant was discarded. The VWP was incubated at 30 °C for 20 h without shaking. In this condition, *E. coli* S17-1 and *C. glutamicum* came into close contact so that conjugation could take place.

After incubation in the VWP, 200 µL of BHI medium was added to each well and the pellet was resuspended by shaking the plate at 1000 rpm for 2 min. Next, the VWP was centrifuged at 4500 rpm and 20 °C for 5 min and 225 µL of the supernatant were discarded. The cell pellet was resuspended in the remaining supernatant by pipetting 40 µL up and down 30 times. From each well, 5 µL was used to create a spot on a BHISG agar plate in SBS-format containing 50 µg mL^{-1} nalidixine and the appropriate selection antibiotic. This procedure was repeated for three separate agar plates, which were then left open for 10 min to dry. The agar plates were incubated at 30 °C for 48 h and stored at 4 °C until further use.

Calculation of transformation efficiency

Analogue to various other authors, the transformation efficiency of electroporation transformations was calculated as the quotient of CFUs and plasmid mass [14-16]. The equation also contains a term that takes into account the deviation between the total volume of a transformation batch and the plated volume.

$$eff = \frac{n_{\mathrm{CFU}}}{m_{\mathrm{DNA}}} \cdot \frac{V_{\mathrm{tot}}}{V_{\mathrm{plate}}} \tag{7}$$

eff: transformation efficiency [ng^{-1}]

n_{CFU}: number of colony forming units [-]

m_{DNA}: mass of plasmid DNA [ng]

V_{tot}: total volume of a transformation batch [mL]

V_{plated}: plated volume [mL]

Additionally, the transformation efficiency of conjugation experiments was calculated as:

$$n_{\mathrm{rel}} = \frac{n_{\mathrm{CFU}}}{OD_{600\ \mathrm{nm}} \cdot V_{\mathrm{plated}}} \tag{8}$$

n_{rel}: number of relative colony forming units [CFU mL^{-1}]

n_{CFU}: number of colonies formed for a specific plated volume [CFU]

OD_{600}: optical density at 600 nm of the *C. glutamicum* culture in the plated volume [-]

V_{plated}: plated volume [mL]

For better comparability between electroporation and conjugation, Eq. 8 was also applied to electroporation experiments.

Table S3: CIDAR MoClo basic parts used in this study.

Addgene Plasmid ID	Plasmid	Part	Description
65980	J23100_AB	Constitutive promoter	High strength
65988	J23103_AB	Constitutive promoter	Low strength
65992	J23106_AB	Constitutive promoter	Medium strength
66024	BCD2_BC	RBS BiCistronic Design	High strength
66032	E0040m (GFP)_CD	Gene of Interest (GoI)	Modified from Bba_E0040 to fix illegal site.
66035	B0015_DE	Terminator	Double terminator (B0010:B0012)
66067	DVK_AE	Backbone Plasmid	Based on pSB1K3

Table S4: Designed and assembled plasmids in this study based on CiDAR MoClo basic part plasmids.

GoI	Name [Backbone, Promotor, RBS, GoI, Terminator]
GFP	DVK_AE_J23100_AB_BCD2_BC_E0040m (GFP)_CD_B0015_DE
GFP	DVK_AE_J23103_AB_BCD2_BC_E0040m (GFP)_CD_B0015_DE
GFP	DVK_AE_J23106_AB_BCD2_BC_E0040m (GFP)_CD_B0015_DE

Table S5: Plasmids pEdit assembled for deletion of *lacZ* gene. For homology region sequences see Table S6.

Edit plasmid	spacer	Homology regions
pEdit 1	spacer1	HR1a+2a - 500 bp
pEdit 2	spacer1	HR1b+2b - 300 bp
pEdit 3	spacer2	HR1a+2a - 500 bp
pEdit 4	spacer2	HR1c+2c - 300 bp
pEdit 5	spacer3	HR1a+2a - 500 bp
pEdit 6	spacer3	HR1c+2d - 300 bp

Table S6: Gene strands and primers used for genome editing of *E. coli*.

Abbreviation	Sequence	Origin
spacer1	agacgaggtctcaggagttgacagctagctcagtcctaggtataatactagtctggggaatgaa<br>tcaggccagttttagagctagaaatagcaagttaaaataaggctagtccgttatcaacttgaaaa<br>agtggcaccgagtcggtgcttttttttactcgagaccgctaat	[17]
spacer2	agacgaggtctcaggagttgacagctagctcagtcctaggtataatactagtctgacaatggca<br>gatcccaggttttagagctagaaatagcaagttaaaataaggctagtccgttatcaacttgaaaa<br>agtggcaccgagtcggtgcttttttttactcgagaccgctagt	CHOPCHOP [18]
spacer3	agacgaggtctcaggagttgacagctagctcagtcctaggtataatactagttcgcacagcgtg<br>taccacaggttttagagctagaaatagcaagttaaaataaggctagtccgttatcaacttgaaaaa<br>gtggcaccgagtcggtgcttttttttactcgagaccgctagt	CHOPCHOP [18]
P1	cgtatcacgaggcagaatttc	This work
P2	cggacaggtatccggtaagc	This work
P3	cgattggctagcccacctgacgtctaagaaaccatt	This work
P4	attaagagatctcctttgagtgagctgataccg	This work
P5	gtcaatggtctcatactaaacggggatactgacg	This work
P6	gtcaatggtctcgcattcacggcgctaatcacgacg	This work
P7	aatcgaggcacacagcgc	This work
P8	gactggagtgccatgtccg	This work
HR1a	atccgtaatcatggtcatagctgtttcctgtgtgaaattgttatccgctcacaatttcacacaacata<br>cgagccggaagcataaagtgtaaagcctggggtgcctaatgagtgagctaactcacattaattg<br>cgttgcgctcactgcccgctttccagtcgggaaacctgtcgtgccagctgcattaatgaatcggc<br>caacgcgcggggagaggcggtttgcgtattgggcgccagggtggtttttcttttcaccagtgag<br>acgggcaacagctgattgcccttcaccgcctggccctgagagagttgcagcaagcggtccac<br>gctggtttgccccagcaggcgaaaatcctgtttgatggtggttaacggcgggatataacatgag<br>ctgtcttcggtatcgtcgtatcccactaccgagatatccgcaccaacgcgcagcccggactcgg<br>taatggcgcgcattgcgcccagcgccatctgatcgttggcaaccagcat	This work
HR1b	tgggtttcaatattggcttcatccaccacatacaggccgtagcggtcgcacagcgtgtaccaca<br>gcggatggttcggataatgcgaacagcgcacggcgttaaagttgttctgcttcatcagcaggat<br>atcctgcaccatcgtctgctcatccatgacctgaccatgcagaggatgatgctcgtgacggttaa<br>cgcctcgaatcagcaacggcttgccgttcagcagcagcagaccattttcaatccgcacctcgc<br>ggaaaccgacatcgcaggcttctgcttcaatcagcgtgccgtcg	This work
HR1c	atccgtaatcatggtcatagctgtttcctgtgtgaaattgttatccgctcacaatttcacacaacata<br>cgagccggaagcataaagtgtaaagcctggggtgcctaatgagtgagctaactcacattaattg<br>cgttgcgctcactgcccgctttccagtcgggaaacctgtcgtgccagctgcattaatgaatcggc<br>caacgcgcggggagaggcggtttgcgtattgggcgccagggtggtttttcttttcaccagtgag<br>acgggcaacagctgattgcccttcaccgcctggccctga	This work
HR2a	aaattactgcgacggctgactttctcaataaatgcctctactgctggcgcaccggcgttaaaaca<br>aaagcctagataaataccaccaacaatcgatcctactaaaatgttgtattgtaacagtggcccga<br>agataaaaataaagaacggcgcaaacatcactaacatgccggtaataatccacagcaggtattt<br>gcgcagcccgagtttgtcagaaagcagaccaaacagcggttggaataatagcgagaacaga<br>gaaatagcggcaaaaataatacccgtatcacttttgctgatatggttgatgtcatgtagccaaatc<br>gggaaaaacgggaagtaggctcccatgataaaaaagtaaaagaaaaagaataaaccgaacat<br>ccaaaagtttgtgtttttaaatagtacataatggatttccttacgcgaaatacgggcagacatggc<br>ctgcccggttattattatttttgacaccagaccaactggtaatggtagcg	This work

Abbreviation	Sequence	Origin
HR2b	aactggtaatggtagcgaccggcgctcagctggaattccgccgatactgacgggctccagga gtcgtcgccaccaatccccatatggaaaccgtcgatattcagccatgtgccttcttccgcgtgca gcagatggcgatggctggtttccatcagttgctgttgactgtagcggctgatgttgaactggaag tcgccgcgccactggtgtgggccataattcaattcgcgcgtcccgcagcgcagaccgttttcgc tcgggaagacgtacggggtatacatgtctgacaatggcagatcc	This work
HR2c	atcggtggccgtggtgtcggctccgccgccttcatactgcaccgggcgggaaggatcgacag atttgatccagcgatacagcgcgtcgtgattagcgccgtggcctgattcattccccagcgacca gatgatcacactcgggtgattacgatcgcgctgcaccattcgcgttacgcgttcgctcatcgccg gtagccagcgcggatcatcggtcagacgattcattggcaccatgccgtgggtttcaatattggct tcatccaccacatacaggccgtagcggtcgcacagcgtgtacca	This work
HR2d	tgtaaacggggatactgacgaaacgcctgccagtatttagcgaaaccgccaagactgttaccc atcgcgtgggcgtattcgcaaaggatcagcgggcgcgtctctccaggtagcgaaagccattttt tgatggaccatttcggcacagccgggaagggctggtcttcatccacgcgcgcgtacatcgggc aaataatatcggtggccgtggtgtcggctccgccgccttcatactgcaccgggcgggaaggat cgacagatttgatccagcgatacagcgcgtcgtgattagcgccgtgg	This work

Table S7: List of further plasmids used for this work.

Plasmid name	Size [kbp]	Key-Features	Origin
pRSET_B_JT	3.5	- pRSET_B backbone<br>- Ampicilin resistance	This work
pTarget	2.1	- Spectinomycin resistance<br>- gRNA	[2]
pEdit_AE	2.8	- Spectinomycin resistance<br>- gRNA<br>- Homologous regions for homology directed repair	This work
pCas	12.5	- Kanamycin resistance<br>- Cas9 production<br>- Inducible λ-Red recombinases production<br>- Inducible gRNA production for pEdit<br>- Temperature sensitive origin of replication	[2]
pEC-T18mob2	6.2	- Tetracycline resistance<br>- Conjugation ready	[19]
pEC-T18mob2_*eYFP_lrp_BB*	7.4	- eYFP production dependent on intracellular L-leucine concentration<br>- Tetracycline resistance<br>- Conjugation ready	[3, 20]
pEC-T18mob2_*ptuf-eYFP*	7.1	- Constitutive eYFP production<br>- Tetracycline resistance<br>- Conjugation ready	This work

References

1. Smaluch, K. et al. Assessing the growth kinetics and stoichiometry of *Escherichia coli* at the single- cell level. *Engineering in Life Sciences* **23**, e2100157 (2023).
2. Jiang, Y. et al. Multigene editing in the *Escherichia coli* genome via the CRISPR-Cas9 system. *Applied and environmental microbiology* **81**, 2506-2514 (2015).
3. Tenhaef, N., Stella, R., Frunzke, J. & Noack, S. Automated Rational Strain Construction Based on High-Throughput Conjugation. *ACS Synthetic Biology* **10**, 589-599 (2021).
4. Hermann, R., Lehmann, M. & Büchs, J. Characterization of gas–liquid mass transfer phenomena in microtiter plates. *Biotechnology and Bioengineering* **81**, 178-186 (2003).
5. Reuss, M. in Recent Advances in Biotechnology. (eds. F. Vardar-Sukan & Ş.S. Sukan) 183-208 (Springer Netherlands, Dordrecht; 1992).
6. Radek, A. et al. Miniaturized and automated adaptive laboratory evolution: Evolving *Corynebacterium glutamicum* towards an improved d-xylose utilization. *Bioresour Technol* **245**, 1377-1385 (2017).
7. Tieleman, D.P. The molecular basis of electroporation. *BMC Biochemistry* **5**, 10 (2004).
8. Tarek, M. Membrane Electroporation: A Molecular Dynamics Simulation. *Biophysical Journal* **88**, 4045-4053 (2005).
9. Matsson, P. & Kihlberg, J. How Big Is Too Big for Cell Permeability? *Journal of Medicinal Chemistry* **60**, 1662-1664 (2017).
10. Ohse, M., Takahashi, K., Kadowaki, Y. & Kusaoke, H. Effects of Plasmid DNA Sizes and Several Other Factors on Transformation of *Bacillus subtilis* ISW1214 with Plasmid DNA by Electroporation. *Bioscience, Biotechnology, and Biochemistry* **59**, 1433-1437 (1995).
11. Hornstein, B.D., Roman, D., Arévalo-Soliz, L.M., Engevik, M.A. & Zechiedrich, L. Effects of Circular DNA Length on Transfection Efficiency by Electroporation into HeLa Cells. *PLoS One* **11**, e0167537 (2016).
12. Molnar, M.J. et al. Factors Influencing the Efficacy, Longevity, and Safety of Electroporation-Assisted Plasmid-Based Gene Transfer into Mouse Muscles. *Molecular Therapy* **10**, 447-455 (2004).
13. Jiang, Y. et al. CRISPR-Cpf1 assisted genome editing of *Corynebacterium glutamicum*. *Nature Communications* **8**, 15179 (2017).
14. Carson, S., Miller, H.B., Witherow, D.S. & Srougi, M.C. in Molecular Biology Techniques (Fourth Edition). (eds. S. Carson, H.B. Miller, D.S. Witherow & M.C. Srougi) 45-52 (Academic Press, 2019).
15. Ruan, Y., Zhu, L. & Li, Q. Improving the electro-transformation efficiency of *Corynebacterium glutamicum* by weakening its cell wall and increasing the cytoplasmic membrane fluidity. *Biotechnology Letters* **37**, 2445-2452 (2015).
16. van der Rest, M.E., Lange, C. & Molenaar, D. A heat shock following electroporation induces highly efficient transformation of *Corynebacterium glutamicum* with xenogeneic plasmid DNA. *Applied Microbiology and Biotechnology* **52**, 541-545 (1999).
17. Zhao, D. et al. Development of a fast and easy method for *Escherichia coli* genome editing with CRISPR/Cas9. *Microbial cell factories* **15**, 1-9 (2016).
18. Labun, K. et al. CHOPCHOP v3: expanding the CRISPR web toolbox beyond genome editing. *Nucleic acids research* **47**, W171-W174 (2019).
19. Tauch, A. et al. Efficient Electrotransformation of *Corynebacterium diphtheriae* with a Mini-Replicon Derived from the *Corynebacterium glutamicum* Plasmid pGA1. *Current Microbiology* **45**, 362-367 (2002).

20. Mustafi, N., Grünberger, A., Kohlheyer, D., Bott, M. & Frunzke, J. The development and application of a single-cell biosensor for the detection of l-methionine and branched-chain amino acids. *Metabolic Engineering* **14**, 449-457 (2012).
